# Differential Regulation of the Surface-Exposed and Secreted SslE Lipoprotein in Extraintestinal Pathogenic *Escherichia coli*

**DOI:** 10.1371/journal.pone.0162391

**Published:** 2016-09-06

**Authors:** Lendl Tan, Danilo G. Moriel, Makrina Totsika, Scott A. Beatson, Mark A. Schembri

**Affiliations:** 1 Australian Infectious Diseases Research Centre, School of Chemistry and Molecular Biosciences, The University of Queensland, QLD 4072, Brisbane, Australia; 2 Institute of Health and Biomedical Innovation, School of Biomedical Sciences, Queensland University of Technology, QLD 4059, Brisbane, Australia; Centre National de la Recherche Scientifique, Aix-Marseille Université, FRANCE

## Abstract

Extra-intestinal pathogenic *Escherichia coli* (ExPEC) are responsible for diverse infections including meningitis, sepsis and urinary tract infections. The alarming rise in anti-microbial resistance amongst ExPEC complicates treatment and has highlighted the need for alternative preventive measures. SslE is a lipoprotein secreted by a dedicated type II secretion system in *E*. *coli* that was first identified as a potential vaccine candidate using reverse genetics. Although the function and protective efficacy of SslE has been studied, the molecular mechanisms that regulate SslE expression remain to be fully elucidated. Here, we show that while the expression of SslE can be detected in *E*. *coli* culture supernatants, different strains express and secrete different amounts of SslE when grown under the same conditions. While the histone-like transcriptional regulator H-NS strongly represses *sslE* at ambient temperatures, the variation in SslE expression at human physiological temperature suggested a more complex mode of regulation. Using a genetic screen to identify novel regulators of *sslE* in the high SslE-expressing strain UTI89, we defined a new role for the nucleoid-associated regulator Fis and the ribosome-binding GTPase TypA as positive regulators of *sslE* transcription. We also showed that Fis-mediated enhancement of *sslE* transcription is dependent on a putative Fis-binding sequence located upstream of the -35 sequence in the core promoter element, and provide evidence to suggest that Fis may work in complex with H-NS to control SslE expression. Overall, this study has defined a new mechanism for *sslE* regulation and increases our understanding of this broadly conserved *E*. *coli* vaccine antigen.

## Introduction

*Escherichia coli* are highly diverse bacteria ranging from harmless gut commensal organisms to specialized pathogens capable of causing a variety of infections in humans and animals [1]. Extra-intestinal pathogenic *E*. *coli* (ExPEC) cause infections outside the intestinal tract, including sepsis, neonatal meningitis and urinary tract infection (UTI). Among these infections, UTIs represent the most substantial burden to healthcare systems worldwide [2, 3].

Uropathogenic *E*. *coli* (UPEC), the primary cause of UTI [4], is the largest and most clinically significant ExPEC pathotype [1, 5]. On a global scale, the prevalence of UPEC (and other *Enterobacteriaceae*) resistant to multiple classes of antibiotics is increasing rapidly [6, 7]. This alarming trend has led to an enhanced rate of UTI treatment failure with conventional antibiotics and increased dependence on last-line therapies such as carbapenems, further promoting the emergence of drug resistant strains [8–11]. Among the potential new approaches currently being investigated for the prevention of UTI, vaccination represents a viable alternative in some patient groups [12, 13]. Strategies for vaccination have included immunization against virulence factors such as adhesins and iron acquisition receptor proteins; as well as the use of lysates of inactivated uropathogens and live attenuated strains [14–19]. Despite these efforts, there is still no vaccine currently available for the prevention of UTI [12, 13].

SslE (also known as YghJ or ECOK1_3385) is a secreted and surface exposed lipoprotein of *E*. *coli* identified by reverse vaccinology as a strongly immunogenic vaccine antigen against ExPEC in a murine sepsis model [20]. SslE is secreted by a dedicated type II secretion system (T2SS) [20, 21], a conserved, multicomponent structure used by Gram-negative bacteria to export a variety of proteins including many virulence factors [22–26]. SslE contributes to biofilm maturation and virulence in enteropathogenic *E*. *coli* (EPEC) [21], although a similar role in atypical EPEC has not been demonstrated [27]. An important function of SslE is its ability to actively degrade intestinal mucins including Muc2, Muc3 and bovine submaxillary mucin, which facilitates *E*. *coli* penetration of mucus and enhances access to apical epithelial cells [28–30]. Immunization with SslE protects mice against both UTI and intestinal infection, suggesting it may be effective as a broadly protective *E*. *coli* vaccine antigen [28]. This was further corroborated by the identification of SslE as an immunogenic antigen in patients infected with enterotoxigenic *E*. *coli* (ETEC) [31, 32].

The molecular mechanisms governing the regulation and expression of SslE by *E*. *coli* strains from different pathotypes remain to be properly elucidated. In ETEC, the *sslE* gene is transcribed as a polycistronic mRNA together with 13 downstream genes encoding its cognate T2SS (*sslE-aspS-pppA-gspC-M*) [33, 34]. The operon is repressed in a temperature-dependent manner by the nucleoid-associated proteins H-NS and StpA, with stronger expression at human physiological temperature (37°C) compared to ambient temperature (22°C) [33]. Both H-NS and StpA bind within the regulatory region of the *sslE* promoter and block transcription initiation by inhibiting promoter open complex formation. StpA binds with higher affinity than H-NS to this region, possibly via the formation of heteromeric complexes with H-NS that enhance its stability [33]. The regulatory role of H-NS in *sslE* transcription in ETEC is consistent with its function as a transcriptional repressor of many virulence genes in UPEC, including genes encoding autotransporter proteins (e.g. UpaC, UpaH, UpaG) [35–37], fimbriae (e.g. F9) [38], and toxins (e.g. α-hemolysin) [39]. In the non-pathogenic *E*. *coli* strain W, SslE expression is influenced by both temperature and nutrients, with stronger expression observed at 37°C in rich medium [40].

In this study, we examined the expression and secretion of SslE in different ExPEC strains. We showed that some strains, including the well-characterized UPEC strain UTI89 and the neonatal meningitis-associated *E*. *coli* (NMEC) strain IHE3034 produce high levels of SslE, suggesting alleviation of H-NS repression at physiological temperature. Consistent with this observation, a genetic screen using the high SslE-expressing strain UTI89 identified the nucleoid associated global transcriptional regulator Fis and the ribosome binding GTPase TypA as positive regulators of *sslE* transcription. Evidence for the function of Fis as an activator of SslE was demonstrated by the generation and analysis of specific mutant and complemented strains and reporter constructs.

## Materials and Methods

### Bacterial strains, plasmids and culture conditions

All strains and plasmids used in this study are listed in Table 1. The UPEC isolates examined for SslE expression were obtained from our in-house clinical collection [41]. *E*. *coli* strains were routinely cultured at 37°C on solid or in liquid Luria-Bertani (LB) media supplemented with appropriate antibiotics (100 μg/ml ampicillin, 100 μg/ml kanamycin, 30 μg/ml chloramphenicol). Where necessary, gene expression was induced with 1mM isopropyl β-D-1-thiogalactopyranoside (IPTG).

**Table 1:** Bacterial strains and plasmids used in this study.

Strain or plasmid	Relevant characteristics	Reference
***E*. *coli* K12 strains**		
MG1655	K-12 reference strain	[42, 43]
BL21 (DE3)	F–ompT hsdSB(rB–, mB–) gal dcm (DE3)	Stratagene
**Clinical UPEC collection**	18 strains randomly selected from a collection of isolates from patients with urosepsis	This study
**ExPEC strains**		
536	UPEC pyelonephritis isolate	[5, 44]
536*fis*	536*fis*::*kan*; Kan^r^	This study
536*fis*(pFis)	536*fis*::*kan* + pFis; Kan^r^ Cm^r^	This study
EC958	ST131 UPEC isolate	[45–47]
EC958*hns*	EC958*hns*::*cm*; Cm^r^	This study
EC958*hns*(pHNS)	EC958*hns* pHNS; Cm^r^	This study
EC958*fis*	EC958*fis*::*cm*; Cm^r^	This study
EC958*fis*(pFis)	EC958*fis* pFis; Cm^r^	This study
IHE3034	NMEC isolate	[20, 48]
IHE3034*sslE*	IHE3034*sslE*::*kan*; Kan^r^	This study
IHE3034*hns*	IHE3034*hns*::*kan*; Kan^r^	This study
IHE3034*hns*(pHNS)	IHE3034*hns*::*kan* pHNS; Kan^r^ Amp^r^	This study
IHE3034*fis*	IHE3034*fis*::*kan*; Kan^r^	This study
IHE3034*fis*(pFis)	IHE3034*fis*::*kan* pFis; Kan^r^ Cm^r^	This study
UTI89	UPEC cystitis isolate	[49, 50]
UTI89*sslE*	UTI89*sslE*::*kan*; Kan^r^	This study
UTI89*hns*	UTI89*hns*::*kan*; Kan^r^	This study
UTI89*hns*(pHNS)	UTI89*hns*::*kan* pHNS; Kan^r^ Amp^r^	This study
UTI89*fis*	UTI89*fis*::*kan*; Kan^r^	This study
UTI89*fis*(pFis)	UTI89*fis*::*kan* pFis; Kan^r^ Cm^r^	This study
UTI89*typA*	UTI89*typA*::*kan*; Kan^r^	This study
UTI89*typA*(pTypA)	UTI89*typA*::*kan* pTypA; Kan^r^ Cm^r^	This study
UTI89*lacI-Z*	UTI89*lacI-Z*::*gfp*	This study
UTI89*lacI-Z sslE*::*lacZ*	UTI89*lacI-Z*::*gfp sslE*::*lacZ*	This study
UTI89*lacI-Z fis*	UTI89*lacI-Z*::*gfp fis*::*kan*; Kan^r^	This study
**Plasmids**		
pKD3	Template plasmid for *cm* gene amplification	[51]
pKD4	Template plasmid for *kan* gene amplification	[51]
pKD46	λ-red recombinase expressing plasmid	[51]
pCP20	FLP expressing plasmid	[52]
pMCSG7	Ligation-independent 6xHis tag cloning vector	[53]
pHNS	pBR322 cloned with *hns* gene	[35]
pSU2718	pACYC184-derived cloning plasmid	[54]
pHNS-cm	pSU2718 cloned with *hns* gene	This study
pTW26	pSU2718 modified with a *xho*I site inserted at original *Hinc*II site	This study
pFis	*dusB-fis* operon cloned in pTW26	This study
pTypA	*typA* gene cloned in pTW26	This study
pQF50	Promoterless *lacZ* reporter plasmid	[55]
pQF50-*sslE*	*sslE* promoter region cloned in pQF50	This study
pQF50-*sslE*M1	pQF50-*sslE* with 2 nucleotide mutations in F1	This study
pQF50-*sslE*M2	pQF50-*sslE* with 3 nucleotide mutations in F2	This study
pQF50-*sslE*M3	pQF50-*sslE* with 5 nucleotide mutations in F1 and F2	This study

### DNA manipulation and genetic techniques

Oligonucleotides were synthesized by Integrated DNA Technologies. A list of primers used in this study is provided in S1 Table. Plasmid DNA was isolated using the QIAprep Spin Miniprep kit (Qiagen). Chromosomal DNA was purified using the Wizard^®^ Genomic DNA Purification Kit (Promega). PCR amplification was performed using One*Taq*^®^ DNA Polymerase (New England Biolabs) or KAPA HiFi^™^ polymerase (Kapa Biosystems) where high-fidelity amplification was required. PCR products and other DNA fragments were purified using the QIAquick PCR Purification kit or the QIAquick Gel Extraction kit (Qiagen). Restriction endonucleases, T4 polynucleotide kinase, T4 ligase and T4 polymerase treatment were performed following manufacturer’s recommendations (New England Biolabs). Nucleic acid quantification was performed using a Nano-Drop 2000 Spectrophotometer (Thermo Scientific). DNA sequencing was performed using Big Dye Terminator Sequencing v3.1 Cycle Sequencing (Applied Biosystems) at the Australian Equine Genomic Research Centre (AEGRC), University of Queensland.

### Construction of plasmids

Plasmids pFis and pTypA were constructed by PCR amplification of *dusB-fis* (primers 4539 and 4538) and *typA* (primers 4535 and 4536), respectively, from UTI89. PCR products were digested with XhoI (forward primer) and HindIII (reverse primer) and ligated into a similarly digested plasmid pTW26 (modified pSU2718 with xhoI) [54]. Two plasmids containing *hns* were used. One contained *hns* cloned in plasmid pBR322 (pHNS) [36]. The second plasmid was constructed by PCR amplification of *hns* (primers 4588 and 4589) from EC958 and ligated into BamHI-HindIII digested plasmid pSU2718 (pHNS-cm). Plasmid pQF50-*sslE* was constructed by PCR amplification of the *sslE* promoter region from the chromosome of UTI89 using primers 5570 and 5571. PCR products were digested with BamHI (forward primer) and HindIII (reverse primer) and ligated into BamHI-HindIII digested plasmid pQF50 [55]. Plasmids pQF50-*sslE*M1, pQF50-*sslE*M2 and pQF30-*sslE*M3 were synthesized by Epoch Life Science Inc.

### Construction of mutants

All mutants were generated using λ-Red recombinase mediated homologous recombination as previously described [51]. Mutant strains IHE3034*sslE*, UTI89*sslE*, UTI89*fis* and UTI89*typA* were constructed using primers with 50-bp homology extensions to amplify the kanamycin (*kan*) resistance cassette with FRT sites from pKD4. The following primers were used. IHE3034*sslE* and UTI89*sslE*: 2886 and 2887; UTI89*fis*: 4411 and 4412; UTI89*typA*: 4399 and 4400. To generate *hns* deletion mutants in strains IHE3034, UTI89 and 536, the *kan* resistance cassette along with 500 bp homology region was amplified from a previously constructed CFT073*hns* mutant [35] using primers 2361 and 2363, generating the strains IHE3034*hns*, UTI89*hns* and 536*hns*. To generate *fis* deletion mutants in IHE3034 and 536, the *kan* resistance cassette with a 250bp homology region was amplified from the UTI89*fis* mutant using primers 6044 and 6043, and λ-Red recombination was used to generate the strains IHE3034*fis* and 536*fis*. To generate deletion mutants in EC958, a three-way PCR procedure was employed to amplify a chloramphenicol (*cm*) resistance cassette with 500bp homology region from pKD3 [46]. EC958*hns* was generated using primers 3915, 3916, 2247, 2248, 3917 and 3918. EC958*fis* was generated using primers 6218, 6219, 6220, 6221, 6222 and 6223. UTI89*fis hns* was generated by mutating *hns* in the UTI89*fis* mutant using a *cm* cassette with 500bp homology region as described above. In order to generate an *sslE-lacZ* reporter construct, the *lacI-Z* genes from UTI89 were initially deleted with a chloramphenicol-*gfp* cassette essentially as previously described [56]; with the exception that the *lacI-Z* genes were deleted instead of the entire *lac* operon; using primers 3997 and 3998. The chloramphenicol resistance cassette was subsequently removed using pCP20 [51], and the *sslE*::*lacZ* promoter fusion strain was generated as previously described [36], with the exception that a chloramphenicol resistance cassette was used instead of zeocin (Primers 4052 and 4061). The chloramphenicol cassette was subsequently removed using pCP20 to generate the strain UTI89*lacI-Z sslE*::*lacZ*.

### Growth assays

All growth assays were performed using the FLUOstar OPTIMA Microplate Reader (BMG LABTECH). Strains were assayed in triplicate in sterile 96-well plates using LB broth as growth media and a total volume of 200 μl. Each starting culture was standardized to OD_600_ = 0.05. Plates were incubated at 37°C with shaking; OD_600_ readings were taken at 15 min intervals.

### Generation of SslE polyclonal antibodies

A segment of the *sslE* gene encoding a 282 amino-acid N-terminal fragment of the protein (minus the predicted signal peptide) was PCR amplified with primers 2673 and 2674 from IHE3034 and cloned as an N-terminal 6xHis fusion in plasmid pMCSG7 via ligation-independent cloning [53]. *E*. *coli* BL21 (DE3) was transformed with this plasmid and cells were grown in LB medium containing IPTG to induce the expression of recombinant SslE. His-tagged recombinant SslE (SslEʹ) was purified using the nickel-nitrilotriacetic acid (Ni-NTA) affinity chromatography (Qiagen) following the manufacturer’s instructions. Purified SslE’ was quantified using the Bicinchoninic Acid Protein Assay Kit (Sigma) and assessed for purity via SDS-PAGE. Polyclonal antibodies were generated in rabbits at the Institute of Medical and Veterinary Science (IMVS), South Australia. The antiserum was adsorbed against a crude protein extract of UTI89*sslE* prior to use.

### Protein preparation and immunoblotting

Strains were cultured in LB to an optical density at 600nm (OD_600nm_) ~3.0. Whole cell lysates were prepared by centrifuging 1 ml of cultures standardized to OD_600nm_ = 1.0. Cell pellets were resuspended in 50 μl water and 50 μl 2×SDS loading buffer (100mM Tris-HCl, 4% w/v SDS, 20% w/v glycerol, 0.2% w/v bromophenol blue, pH 6.8). Supernatant proteins were prepared following a standard procedure, which involved centrifugation of an OD_600nm_ ~3.0 culture, filtration of the supernatant fraction (0.22μm filter) and precipitation with 10% trichloroacetic acid (TCA; 7.2 ml filtered supernatant, 0.8 ml 100% TCA). Precipitated proteins were pelleted by centrifugation, washed twice in 100% ethanol and air-dried. Proteins were resuspended in 50 μl of resuspension buffer (50 mM Ammonium Bicarbonate, 3 M Urea, 5 mM DTT) and an equal volume of 2×SDS loading buffer and samples were boiled for 10 min prior to electrophoresis; a volume of 10 μl was routinely analyzed. SDS-PAGE and transfer of proteins to a PVDF membrane for western blot analysis was performed as previously described [57]. SslE polyclonal antibodies were used as the primary antibody, and alkaline phosphatase conjugated anti-rabbit antibodies (Sigma Aldrich) were used as the secondary antibody. SIGMAFAST^™^ BCIP^®^/NBT (Sigma-Aldrich) was used as the substrate for detection. Western blots were scanned using the Bio-Rad GS-800^™^ calibrated imaging densitometer.

### Transposon mutagenesis

Transposon mutagenesis of UTI89*lacI-Z sslE*::*lacZ* was performed using the Epicentre EZ::Tn*5* custom transposome construction kit. A miniTn*5*-Cm transposon was generated by PCR using plasmid pKD3 as template DNA with primers 2279 and 2280 that contain Tn*5* mosaic ends. Purified Tn*5*-Cm DNA was phosphorylated and incubated with the Transposase (1μg DNA with 4U Transposase). Transposomes were transformed into competent UTI89*lacI-Z sslE*::*lacZ* cells via electroporation and transposon mutants were plated on LB agar supplemented with chloramphenicol and 5-bromo-4-chloro-3-indolyl β-_D_-galactopyranoside (X-Gal). Transposon mutants with altered β-galactosidase activity (determined via blue-white screening) were selected and confirmed by repeat subculture. The insertion site of the transposon mutants was identified via a 2-step arbitrary PCR as previously described [58], with the following primers specific to the chloramphenicol cassette on the transposon: 2209 (round 1) and 3340 (round 2).

### 5′ Rapid amplification of cDNA ends (5′ RACE)

The transcription start site of *sslE* was determined using 5′ RACE (Version 2.0; Invitrogen) [59]. Exponentially growing cells (OD_600nm_ = 0.6) were stabilized with two-volumes of RNAprotect Bacteria Reagent (Qiagen) prior to RNA extraction using the RNeasy Mini Kit (Qiagen) with optional on-column DNase digestion. First-strand cDNA was synthesized and PCR amplified using the following gene specific primers: 4207, 4208 and 4209 following manufacturer’s specification. Amplified cDNA ends were sequenced to determine the transcription start site.

### Quantitative reverse transcription PCR (qRT-PCR)

Total RNA extraction was performed as described above. Purified RNA samples were further treated with rDNase I (Ambion) to ensure the complete removal of contaminating DNA, and re-purified using the RNeasy Mini Kit (Qiagen) RNA cleanup protocol. First-strand cDNA synthesis was performed using the SuperScript^®^ III First-Strand Synthesis System (Invitrogen) as per manufacturer’s recommendation. Real-time PCR was performed using SYBR^®^ Green PCR Master Mix (Applied Biosystems) on a ViiA^™^ 7 Real-Time PCR System (Applied Biosystems), using the following primers for *sslE*, primers 4205 and 4206. Transcript levels of each gene were normalized to *gapA* as the endogenous gene control (primers 820 and 821). Gene expression levels were determined using the 2^-ΔΔCT^ method [60], with relative mRNA fold-difference expressed against the respective wild-type strains. All experiments were performed as three independent replicates, with all samples analyzed in triplicate. Statistical analysis of fold differences from wild-type, mutant and complemented strains was performed using an unpaired, two tailed student’s t-test.

### β-galactosidase assay

β-galactosidase assays were performed essentially as previously described [61]. Briefly, strains to be assessed were grown overnight in LB broth supplemented with the necessary antibiotics. Cultures were diluted in Z buffer (60 mM Na_2_HPO_4_, 40 mM NaH_2_PO_4_, 50 mM β-mercaptoethanol, 10 mM KCl, 1mM MgSO_4_, pH 7) with 0.004% SDS and chloroform added. Samples were vortexed and incubated at 28°C to permeabilize the cells. The substrate o-nitrophenyl- β-_D_-galactopyranoside (ONPG) was added to initiate the reaction which was subsequently stopped with sodium bicarbonate. β-galactosidase activity was assessed in quadruplicate for each strain by measuring the absorbance at 420 nm. All experiments were performed as three independent replicates. Statistical analysis of β-galactosidase levels between each wild-type and *fis* mutant strains carrying the different pQF50 constructs was performed using an unpaired, two tailed student’s t-test.

## Results

### SslE is expressed at different levels by diverse ExPEC strains

**Fig 1:**
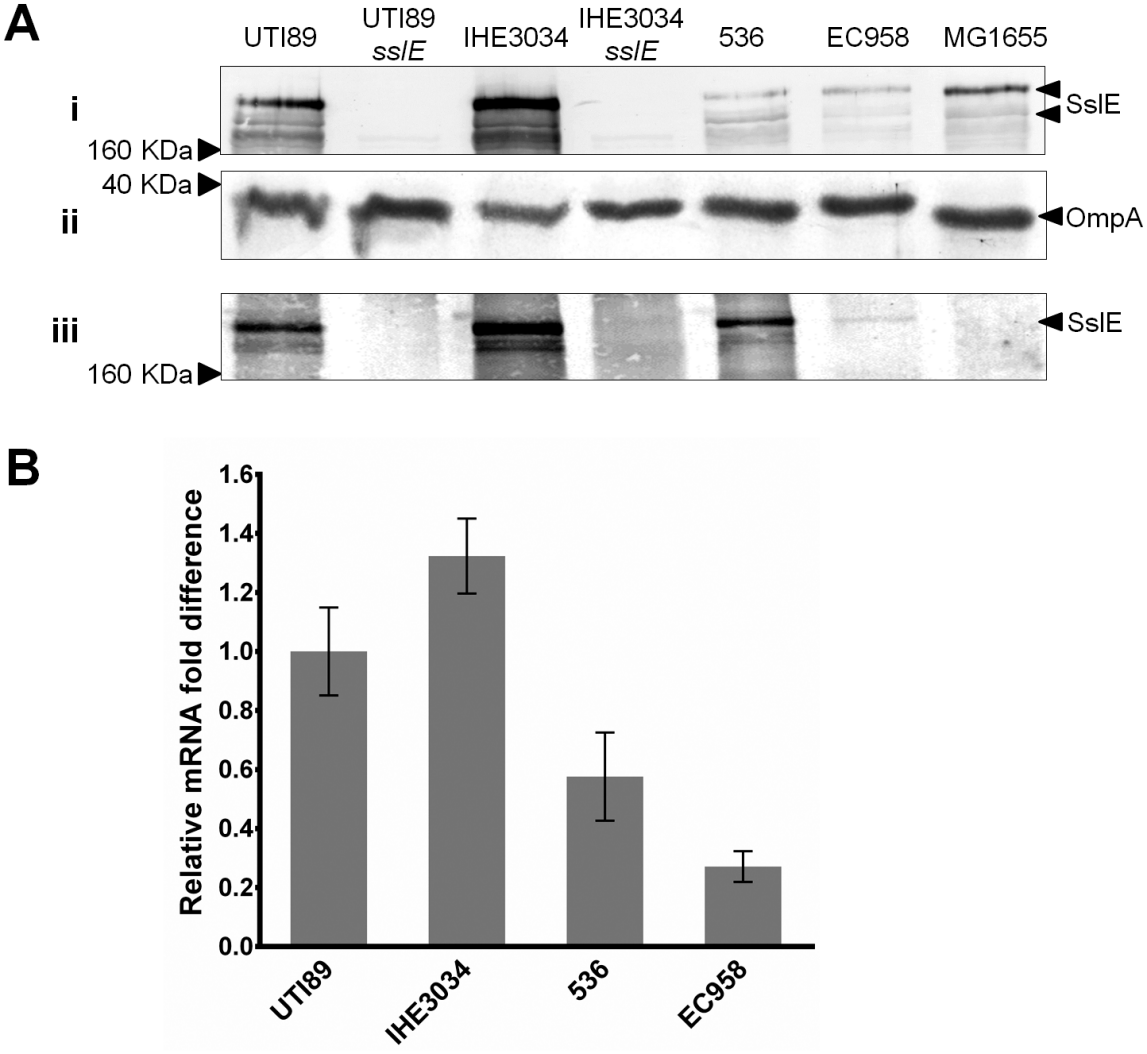
Western blot and qRT-PCR analysis to examine *sslE* expression in ExPEC strains. (A) Western blot analysis of SslE from (i) whole-cell lysates and (iii) supernatant fractions prepared from UTI89, UTI89*sslE*, IHE3034, IHE3034*sslE*, 536, EC958 and MG1655. SslE has a predicted size of approximately 167 kDa. Two major cross-reacting bands of this size were detected (indicated by arrows), possibly representing full-length and processed SslE. No cross-reacting bands were detected in samples prepared from UTI89*sslE* and IHE3034*sslE*, demonstrating the specificity of antibody. (ii) Loading control for whole cell lysate samples. The same samples used above were examined by western blot using an OmpA antibody. Similar levels of OmpA were detected in all samples, indicating equivalent loading of total protein. (B) Relative fold-difference of *sslE* transcript levels of ExPEC strains UTI89, IHE3034, 536, EC958 as determined by qRT-PCR. All mRNA levels were calculated relative to the level of UTI89 *sslE* mRNA. The relative *sslE* mRNA levels were consistent with the data observed from the western blot analysis. The data was obtained from three independent experiments; error bars indicate standard deviation.

The expression of SslE was assessed by western blot analysis using an SslE-specific antibody. SslE expression was examined in the whole-cell lysate and supernatant fractions prepared from cultures of four well-characterized reference ExPEC strains grown at 37°C: UTI89, EC958 (cystitis isolates), IHE3034 (NMEC isolate), and 536 (pyelonephritis isolate) (Table 1; Fig 1A). A band corresponding to the size of SslE was detected in both whole-cell and supernatant fractions prepared from all four strains. However, different levels of SslE expression were observed based on calibration with an OmpA loading control, with the strongest expression detected in IHE3034 and UTI89. To confirm the identity of the SslE protein band, the *sslE* gene was deleted in UTI89 and IHE3034 to generate the mutant strains UTI89*sslE* and IHE3034*sslE*; protein preparations from both mutants did not react with the SslE antibody. SslE is a large ~167 kDa protein and we consistently observed a band of this size as well as smaller cross-reacting bands in our western blot analysis; these smaller bands are likely to represent SslE breakdown products as they were absent from UTI89*sslE* and IHE3034*sslE*. SslE expression was also examined in the K-12 strain MG1655, which possesses an intact *sslE* gene but a mutated downstream T2SS locus. Consistent with previous reports [20], SslE expression was detected in the whole-cell lysate of MG1655 but not in the secreted fraction. Based on the western blot analysis of both whole-cell and supernatant fractions, we observed an overall correlation between the level of SslE synthesis and secretion, a finding consistent with the fact that the *sslE* gene is co-transcribed as a single operon with its cognate T2SS encoding genes [33]. We also examined SslE secretion in 18 UPEC strains from our clinical isolate collection that were positive for the *sslE* and T2SS-encoding genes. SslE was detected in the supernatant fraction of all 18 isolates, however consistent with our results for UTI89, EC958, IHE3034 and 536, different levels of SslE secretion were observed among these strains (S1 Fig).

### SslE expression is regulated at the level of transcription

The transcript abundance of *sslE* was assessed in UTI89, IHE3034, EC958 and 536 via qRT-PCR (Fig 1B). Compared to UTI89, *sslE* transcript level was higher in IHE3034 (~1.3 fold increase), and lower in 536 (~1.7-fold decrease) and in EC958 (~3.7-fold decrease), respectively. Overall, the difference in relative mRNA transcript level of *sslE* for each strain corresponded with the expression pattern observed by western blot analysis. Taken together, the results suggest that the expression of SslE is regulated at the transcriptional level.

### H-NS is not a strong repressor of *sslE* transcription in UTI89 at 37°C

**Fig 2:**
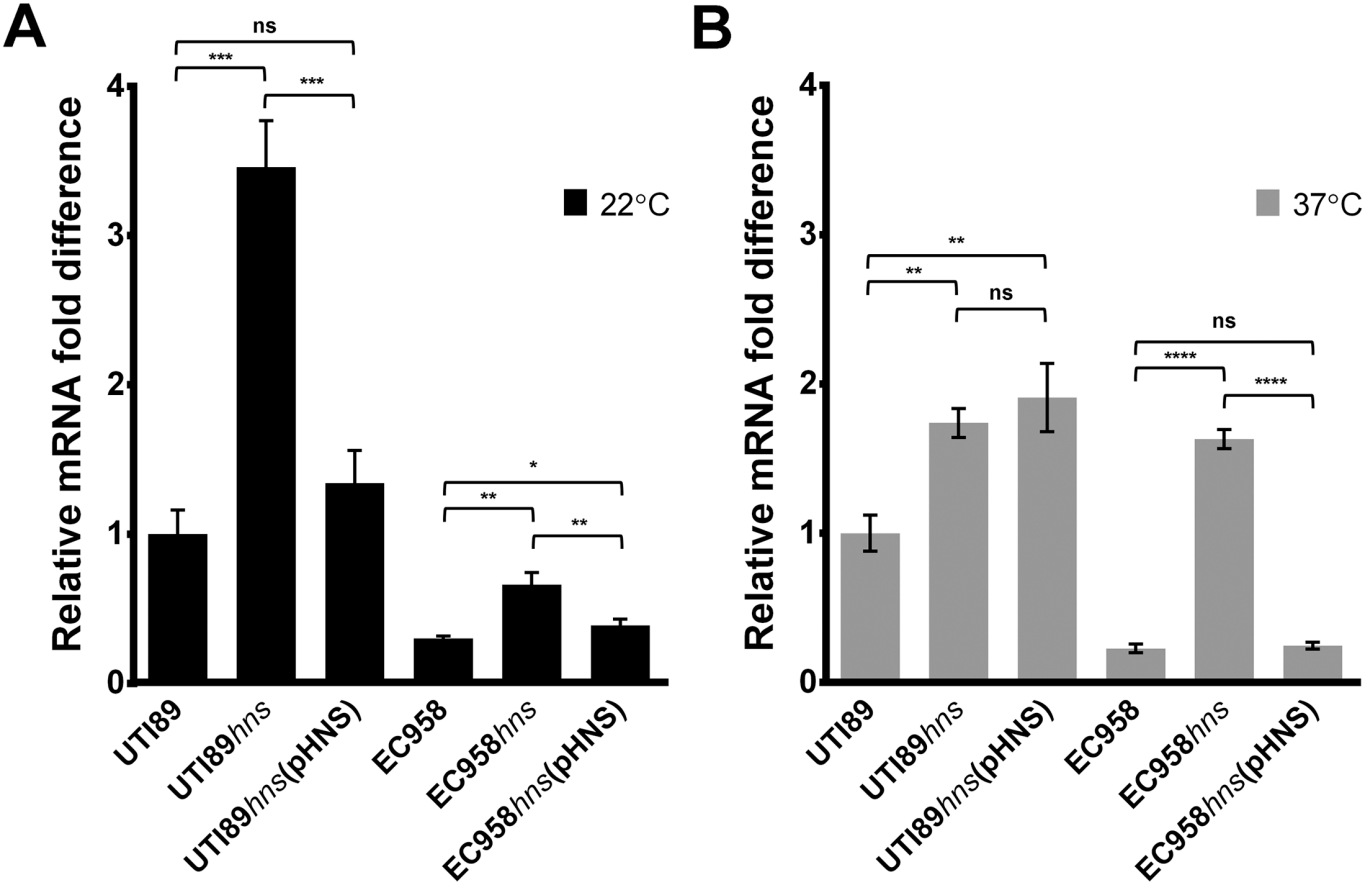
Effect of *hns* deletion on *sslE* transcription in UTI89 and EC958 at 22°C and 37°C via qRT-PCR analysis. Relative fold-difference of *sslE* transcript levels of UTI89 and EC958 with their respective *hns* mutant and complemented strains grown at (A) 22°C (black bars) and (B) 37°C (grey bars). All mRNA levels were calculated relative to the level of UTI89 *sslE* mRNA at the respective temperature. The data was obtained from three independent experiments; error bars indicate standard deviation. Statistical analysis was performed using an unpaired, two-tailed t-test.

In ETEC, H-NS mediates temperature-dependent repression of *sslE* transcription [33]. To investigate the impact of H-NS on SslE expression in UPEC, the *hns* gene was mutated in the high SslE-expressing strain UTI89 (UTI89*hns*) and the low SslE-expressing strain EC958 (EC958*hns*). In both strains, the mutation of *hns* had a similar effect and resulted in slightly reduced growth (S2 Fig). The deletion mutants were complemented with a recombinant plasmid containing the *hns* gene (pHNS or pHNS-cm, respectively). The wild-type, *hns* mutant and complemented strains were grown at 22°C and 37°C, and the level of *sslE* transcription was assessed by qRT-PCR (Fig 2). Consistent with previous data from ETEC H10407 [33], the transcript level of *sslE* was strongly increased in UTI89*hns* (~3.5-fold) and EC958*hns* (~2.2-fold) at 22°C and was reduced to wild-type levels in the complemented strains UTI89*hns*(pHNS) and EC958*hns*(pHNS-cm). At 37°C, the *sslE* transcript level in EC958*hns* increased ~7.2-fold and was restored to wild-type level in EC958*hns*(pHNS-cm). In contrast, however, the increase in *sslE* transcript level in UTI89*hns* at 37°C was smaller (~1.7-fold), and the effect of H-NS could not be complemented in UTI89*hns*(pHNS). A similar difference in SslE expression in UTI89 (compared to EC958) was also observed by western blot analysis (S3 Fig). Overall, the phenotype of the UTI89*hns* and UTI89*hns*(pHNS) strains, together with the high expression of SslE in UTI89 at 37°C (Fig 1), led us to investigate the regulation of *sslE* at this temperature as outlined in the experiments described below.

### Identification of genes involved in the positive regulation of *sslE*

To investigate the genetic basis of strong *sslE* transcription in UTI89, we generated a chromosomal *sslE* promoter-*lacZ* reporter fusion construct. We first mapped the transcription start site and promoter of *sslE* in UTI89, which was in agreement to the promoter mapped in ETEC H10407 [33]. A *lacZ* reporter was inserted as a transcriptional fusion to the *sslE* promoter on the chromosome of a UTI89*lacI-Z* strain to generate the strain UTI89*lacI-Z sslE*::*lacZ*. When grown on LB agar in the presence of X-gal at 37°C, all UTI89*lacI-Z sslE*::*lacZ* colonies were dark blue, indicating strong activity of the *sslE* promoter. In order to identify transcriptional regulators of *sslE* in UTI89, the reporter strain UTI89*lacI-Z sslE*::*lacZ* was subjected to transposon mutagenesis using a mini-Tn*5* cassette. Approximately 25,000 transposon mutants (~5-fold coverage) were generated and screened on LB agar supplemented with chloramphenicol and X-gal. In this screen, 13 mutants were identified that grew as white/pale-blue colonies. The β-galactosidase activity of the 13 transposon mutants was measured (S2 Table) and the transposon insertion site determined by arbitrary PCR. Overall, Tn*5* insertions were identified within six different genes, three of which contained multiple independent insertions; two mutants contained insertions in the *lacZ* gene (Table 2). The three genes with more than one independent Tn*5* insertions were: (i) *fis*, which encodes a nucleoid associated protein that acts as a global transcriptional regulator [62–66], (ii) *typA* (also known as *bipA*) which encodes a ribosome associated GTPase that has been associated with post-transcriptional regulation in EPEC [67, 68], and (iii) *nusA* which encodes a co-factor of Rho-dependent transcriptional termination [69, 70]. In addition, two mutants containing a unique Tn*5* insertion in the tandemly arranged *ygaZ-ygaH* genes, respectively, were identified; the function of these genes is not known. Finally, one mutant contained a Tn*5* insertion in *dusB* (previously known as *yhdG*) [71, 72], which encodes for a tRNA-dihydrouridine synthase [73]. The *dusB* gene is located immediately upstream of *fis*; the two genes are co-transcribed as a bicistronic operon [74, 75] and DusB is absolutely required for efficient translation of *fis* mRNA [72, 76]. Based on these findings, we selected Fis and TypA for further analysis as potential regulators of *sslE* transcription.

**Table 2:** List of genes identified in the transposon mutagenesis screen.

Gene	Description	Number independent insertions
*typA*	Ribosome associated GTPase	4
*fis*^a^	Nucleoid-associated transcriptional factor	2
*nusA*	Rho-dependent transcriptional termination co-factor	2
*ygaZ*^b^	Hypothetical protein of unknown function	1
*ygaH*^b^	Hypothetical protein of unknown function	1
*dusB*^a^	tRNA-dihydrouridine synthase	1

^a,b^Tandemly arranged genes

### The nucleoid associated protein Fis and ribosome binding GTPase TypA are positive regulators of SslE expression

**Fig 3:**
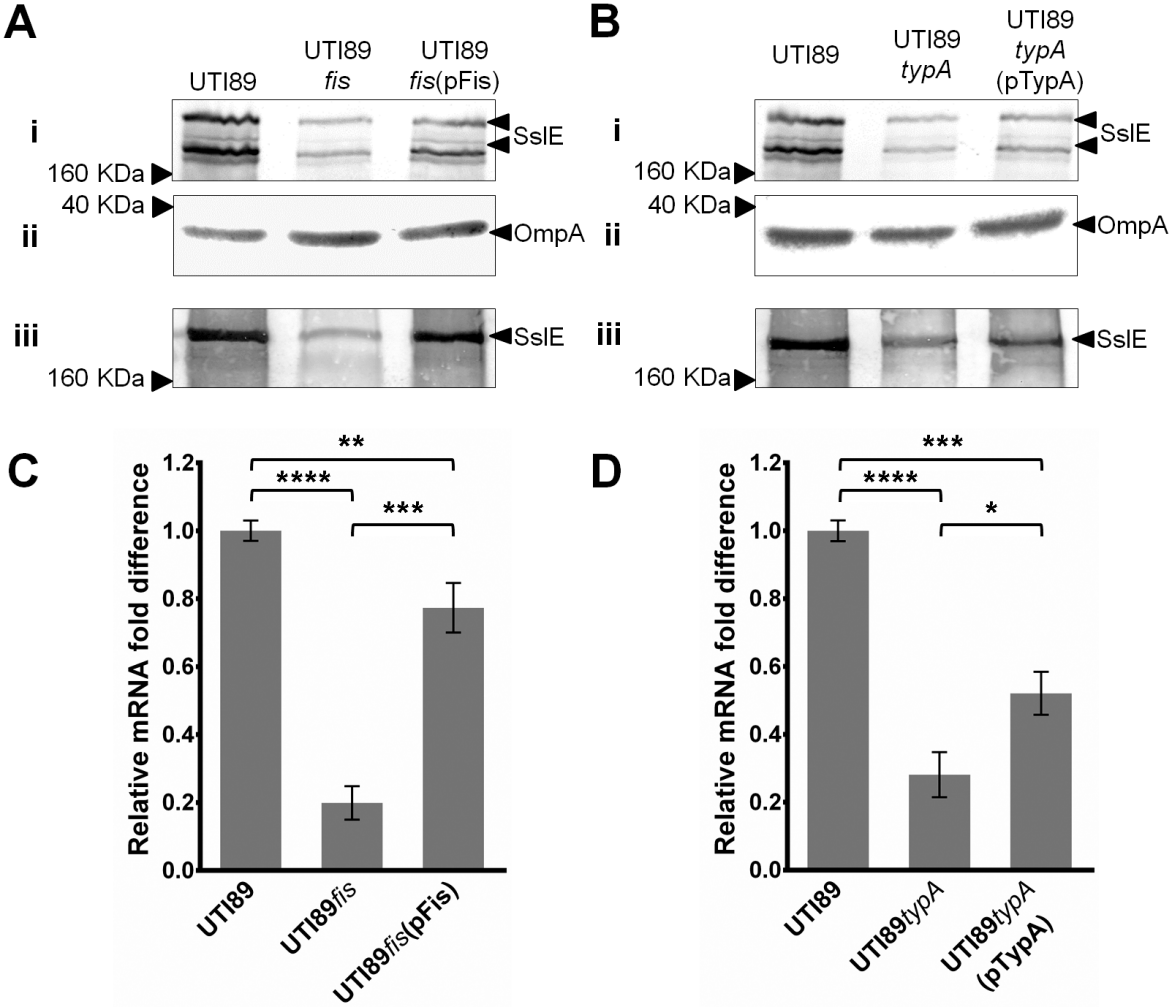
Effect of *fis* and *typA* deletion in UTI89 on *sslE* expression determined via western blot and qRT-PCR analysis (A) Western blot analysis of SslE using (i) whole-cell lysates and (iii) supernatant fractions prepared from UTI89, UTI89*fis* and UTI89*fis*(pFis). (ii) Western blot loading control for whole cell lysate samples using an OmpA antibody. (B) Western blot analysis of SslE using whole-cell lysates and supernatant fractions prepared from UTI89, UTI89*typA* and UTI89*typA*(pTypA). (ii) Western blot loading control for whole cell lysate samples using an OmpA antibody. (C) Relative fold-difference of *sslE* transcript levels of UTI89, UTI89*fis* and UTI89*fis*(pFis); mRNA levels were calculated relative to the level of UTI89 *sslE* mRNA. (D) Relative fold-difference of *sslE* transcript levels of UTI89, UTI89*typA* and UTI89*typA*(pTypA); mRNA levels were calculated relative to the level of UTI89 *sslE* mRNA. The relative *sslE* mRNA levels were consistent with the data observed from the western blot analysis. The data was obtained from three independent experiments; error bars indicate standard deviation. Statistical analysis was performed using an unpaired, two-tailed t-test.

The *fis* and *typA* genes were mutated in UTI89 to generate strains UTI89*fis* and UTI89*typA*. The expression of SslE was then examined by western blot analysis of whole-cell lysates and supernatant fractions prepared from wild-type UTI89, UTI89*fis* and UTI89*typA* following growth in LB broth at 37°C (Fig 3A and 3B). A strong reduction in the level of SslE was detected in preparations from UTI89*fis*. A reduction in SslE expression was also observed in UTI89*typA*, albeit not as strong. To confirm the role of *fis* and *typA* in the regulation of *sslE*, the mutants were complemented with a plasmid containing each respective gene (i.e. pFis and pTypA). Complementation of UTI89*fis* with plasmid pFis restored the amount of SslE detected in whole-cell lysates and supernatant fractions to wild-type level (Fig 3A). In the case of the UTI89*typA* mutant, transformation with plasmid pTypA resulted in partial complementation of SslE expression (Fig 3B). Taken together, these results confirm a role for Fis and TypA in *sslE* regulation.

### Fis and TypA affect the transcription of *sslE*

We sought to investigate if the regulatory effect of Fis and TypA occurs at the transcriptional level. Relative mRNA transcript levels of *sslE* were determined via qRT-PCR using wild-type UTI89 and the respective *fis* and *typA* mutant and complemented strains following growth in LB broth at 37°C (Fig 3C). The transcript level of *sslE* was reduced ~5-fold compared to wild-type UTI89. In the complemented UTI89*fis*(pFis) strain, the *sslE* transcript level was restored to almost wild-type level. The transcript level of *sslE* was also decreased in UTI89*typA* (~3.6-fold) and partially complemented in UTI89*typA*(pTypA); this pattern was similar to that observed by western blot analysis. Taken together, the agreement of the qRT-PCR analysis findings with the levels of SslE protein expression detected by western blot analysis indicates that both Fis and TypA affect *sslE* transcription. Fis is global transcriptional factor that regulates genes involved in multiple processes including virulence, metabolism and DNA replication [62, 64–66, 77]. TypA, on the other hand, is a ribosome binding GTPase that has been shown to contribute to the regulation of several virulence genes [68, 78–80]. The precise mechanism by which TypA exerts its regulatory effect remains unclear, however current evidence suggests this occurs at the level of translation [67, 68, 81, 82]. The finding that TypA affected the transcript levels of *sslE* suggests that its regulatory effect might be indirect. The remainder of our study focused on the characterization of Fis as a transcriptional activator of *sslE* in UTI89.

### Mutation of a putative Fis-binding site within the *sslE* promoter alters its activity

**Fig 4:**
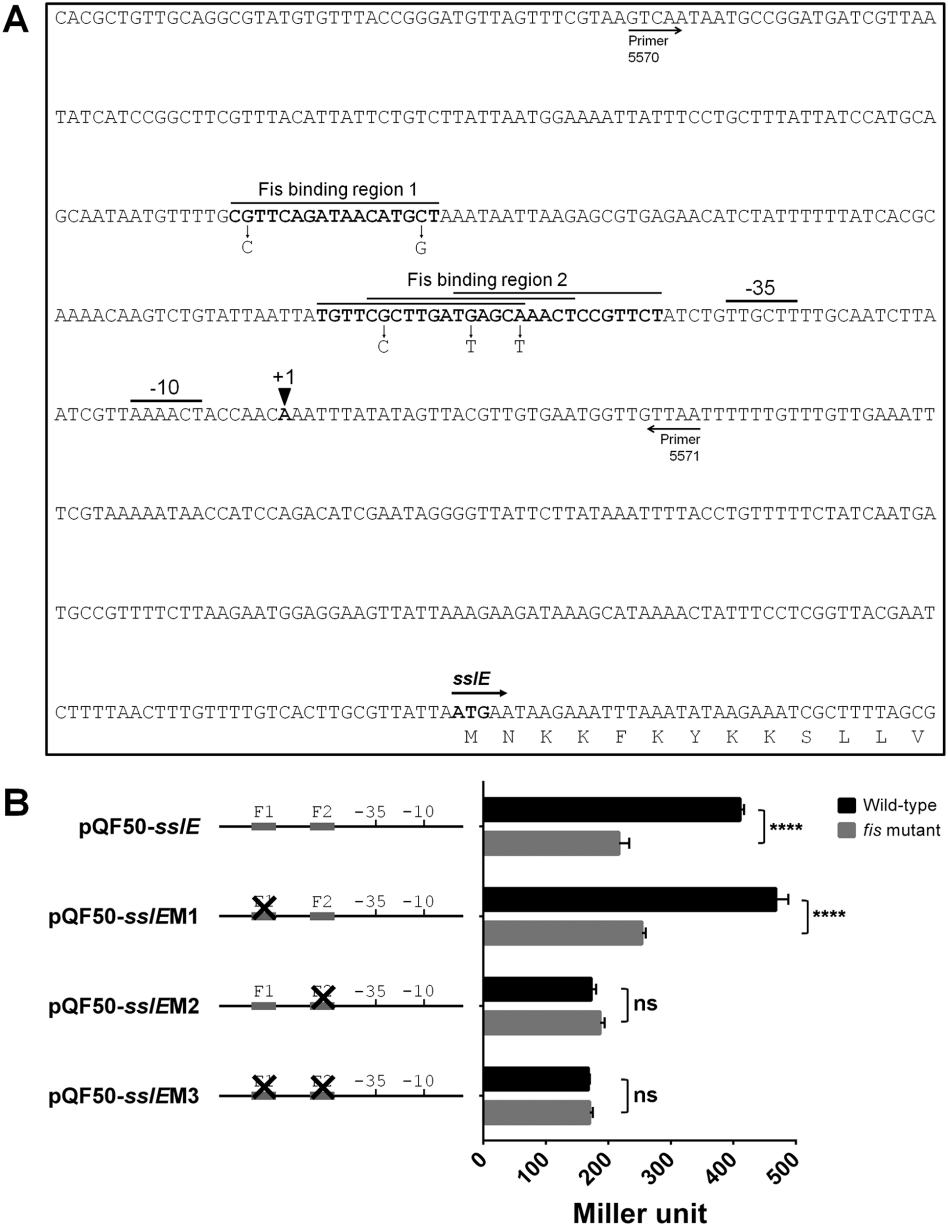
Promoter region of *sslE* in UTI89 and analysis of the effect of putative Fis-binding sequences on *sslE* promoter activity (A) Promoter region of *sslE* in UTI89. The transcription start site as determined via 5'RACE is indicated as +1. Putative -10 and -35 promoter sites and the ATG translational start site are indicated accordingly. The region cloned into the promoter-less *lacZ* reporter plasmid pQF50 is indicated by arrows showing the binding positions of the primers used to amplify the fragment. Putative Fis-binding sites are indicated in bold and overlined. Fis-binding region 1 (F1) consists of one Fis consensus binding sequence. Fis-binding region 2 (F2) consists of three over-lapping consensus sequences. The nucleotide changes introduced to disrupt the Fis-binding sites in the constructs are indicated by an arrow depicting the nucleotide change. (B) β-galactosidase activity represented as Miller units measured for the various *sslE* promoter-*lacZ* reporter plasmid constructs in UTI89*lacI-Z* (wild-type) and UTI89*lacI-Z fis*:*kan* (*fis* mutant) strains. Plasmid pQF50-*sslE* containing the intact *sslE* promoter resulted in approximately double the β-galactosidase activity in the wild-type compared to the *fis* mutant (p < 0.05; t-test). Plasmid pQF50-*sslE*M1 containing mutations in F1 resulted in similar levels of β-galactosidase activity to pQF50-*sslE* in both strains. Plasmids pQF50-*sslE*M2 and pQF50-*sslE*M3, which were disrupted in F2 and F1/F2, respectively, resulted in a similarly reduced β-galactosidase activity in both the wild-type and the *fis* mutant strains (not significant; ns), indicating that F2 is important for Fis activation of the *sslE* promoter.

The binding dynamics of Fis to DNA and its role as a transcriptional regulator have been extensively studied [83, 84]. The *sslE* promoter element contains two regions that contain putative Fis-binding consensus sequences based on a 15 bp sequence inclusion/exclusion rule for Fis binding as determined by previous studies [83, 84]. The first putative Fis-binding site is centered at position -138 (Fis-binding region 1; F1), while the second region consists of three overlapping putative Fis-binding sequences proximal to the -35 element, between positions -41 to -68 (Fis-binding region 2; F2) (Fig 4A). To examine the role of these putative Fis-binding sites on *sslE* promoter activity, the *sslE* promoter region was cloned into the *lacZ* reporter plasmid pQF50 to generate pQF50-*sslE*. Three additional constructs were created to introduce point mutations in key nucleotides within the putative Fis-binding sites (Fig 4A). The first construct, pQF50-*sslE*M1, contained two nucleotide changes that disrupted F1; the second construct, pQF50-*sslE*M2, contained three nucleotide changes that disrupted all three overlapping putative Fis binding sequences within F2; while the last construct, pQF50-*sslE*M3, contained both sets of mutations. All four plasmid constructs were transformed into UTI89*lacI-Z* (wild-type) and UTI89*lacI-Z fis* (*fis* mutant) and the promoter activity in each strain was quantified by the measurement of β-galactosidase activity (Fig 4B). The β-galactosidase activity measured from UTI89*lacI-Z* containing pQF50-*sslE* (410.8±6.3 Miller units) was approximately double the activity measured from UTI89*lacI-Z fis* containing pQF50-*sslE* (217±16.2 Miller units), confirming that Fis positively regulates the activity of the *sslE* promoter. The β-galactosidase activity derived from plasmid pQF50-*sslE*M1 (contains mutations in F1) was similar to that derived from pQF50-*sslE* in both strain backgrounds, indicating that F1 is not required for Fis activation of the *sslE* promoter. In contrast, the β-galactosidase activity derived from plasmid pQF50-*sslE*M2 (contains mutations in F2) was significantly reduced in UTI89*lacI-Z* (172.7±7.8 Miller units), and similar to that obtained in UTI89*lacI-Z fis* (189.7±8 Miller units). The β-galactosidase activity derived from plasmid pQF50-*sslE*M3 (contains mutations in F1 and F2) was similar to that derived from pQF50-*sslE*M2 in wild-type and *fis* mutant backgrounds. Overall, these data suggest that F2 is important for activation of the *sslE* promoter by Fis, and that disruption of this putative binding region is sufficient to abolish Fis activation of the *sslE* promoter.

### Fis positively regulates *sslE* transcription and expression in the other ExPEC strains

**Fig 5:**
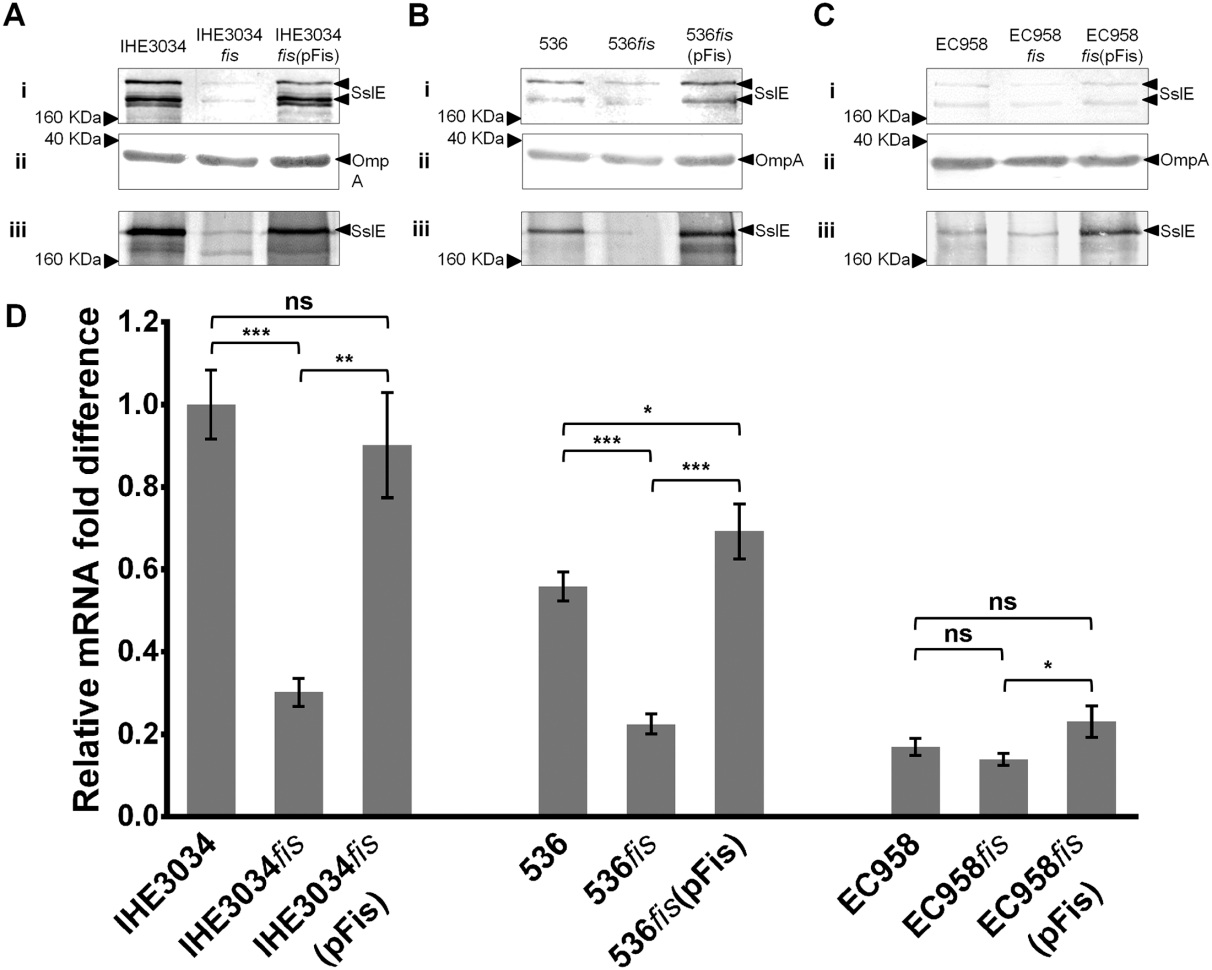
Effect of *fis* deletion on *sslE* expression in strains IHE3034, 536 and EC958 determined via western blot and qRT-PCR analysis. Western blot analysis of SslE using preparations from (A) IHE3034, (B) 536 and (C) EC958; each with their respective *fis* mutant and complemented strains. In each analysis, (i) whole-cell lysates and (iii) supernatant fractions were examined. In addition, (ii) a control for whole cell lysate samples was performed using an OmpA antibody. (D) Relative fold-difference of *sslE* transcript levels of IHE3034, 536 and EC958, and their respective *fis* mutant and complemented strains. All mRNA levels were calculated relative to the level of IHE3034 *sslE* mRNA. The relative *sslE* mRNA levels were consistent with the data observed from the western blot analysis. The data was obtained from three independent experiments; error bars indicate standard deviation. Statistical analysis was performed using an unpaired, two-tailed t-test.

To examine the effect of Fis on SslE expression in IHE3034, 536 and EC958, the *fis* gene was mutated in all three strains and similarly complemented with the plasmid pFis. SslE expression and transcription was examined via western blot analysis and qRT-PCR (Fig 5). For IHE3034, the phenotype observed in the *fis* mutant and complemented strains was similar to that observed for UTI89 (Fig 5A). In 536, SslE expression and secretion was reduced in 536*fis*, and complemented to levels higher than wild-type in 536*fis*(pFis) (Fig 5B). In EC958, due to the lower level of SslE expression, the effect of Fis was not as clear. However, as observed in 536, over-expression of Fis (from plasmid pFis) increased SslE expression in EC958 (Fig 5C). In all cases, relative *sslE* transcript levels as determined via qRT-PCR showed an overall pattern consistent with data from the western blot analysis (Fig 5D). Taken together, our data demonstrates a role for Fis in the activation of *sslE* transcription in multiple *E*. *coli* strains.

### Fis and H-NS have contrasting roles in *sslE* regulation

**Fig 6:**
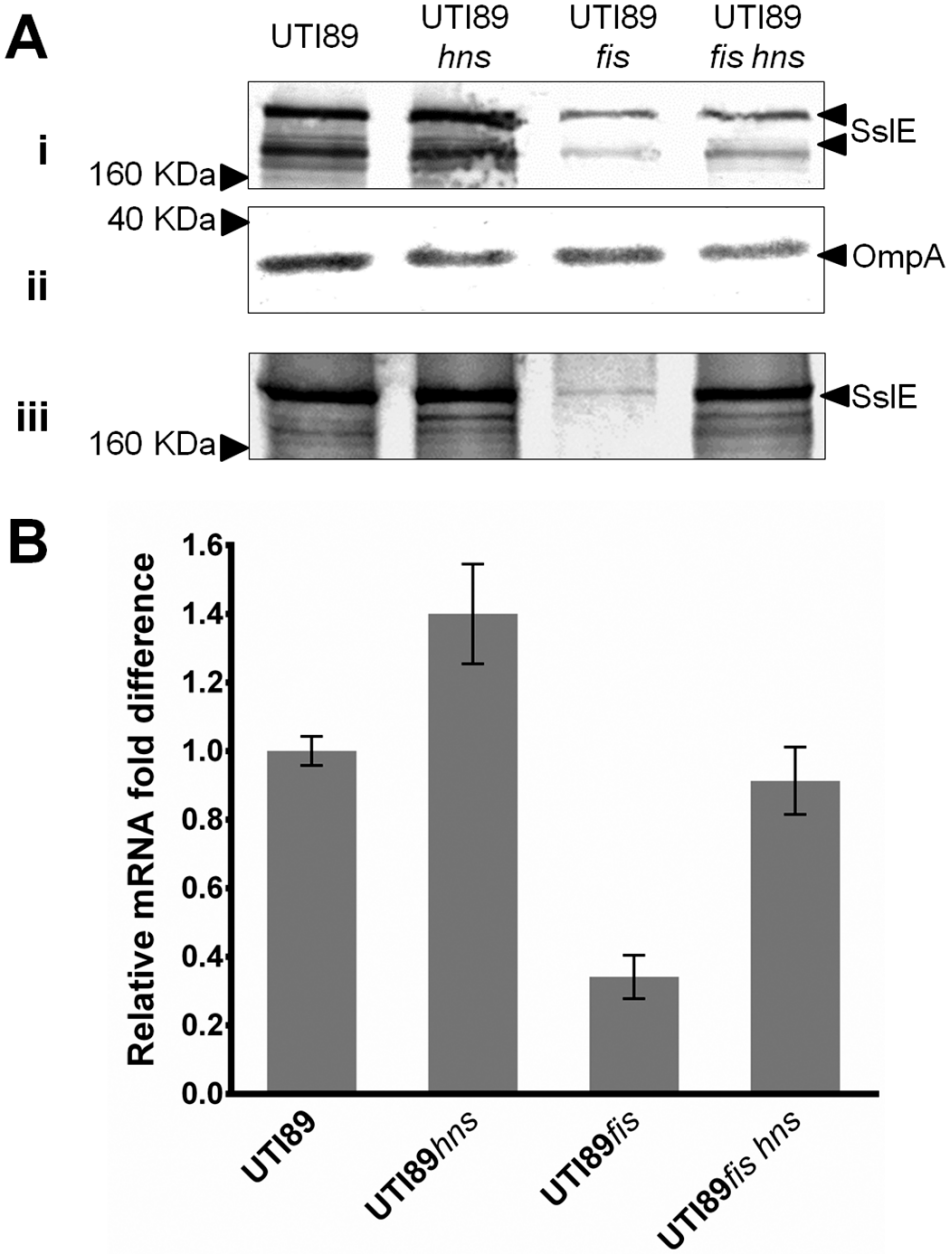
Effect of *fis* and *hns* double deletion on *sslE* expression in UTI89 determined via western blot and qRT-PCR analysis. (A) Western blot analysis of SslE using (i) whole-cell lysates and (iii) supernatant fractions prepared from UTI89, UTI89*hns*, UTI89*fis* and UTI89*fis hns*. (ii) Western blot loading control for whole cell lysate samples using an OmpA antibody. The overall level of SslE was reduced in UTI89*fis hns* compared to wild-type UTI89. (B) Relative fold-difference of *sslE* transcript levels of UTI89, UTI89*hns*, UTI89*fis* and UTI89*fis hns*. All mRNA levels were calculated relative to the level of UTI89 *sslE* mRNA. The relative *sslE* mRNA levels were consistent with the data observed from the western blot analysis. The data was obtained from three independent experiments; error bars indicate standard deviation.

To investigate whether the effect of Fis on SslE expression in UTI89 is dependent on H-NS, a *fis-hns* double mutant was generated (UTI89*fis hns*), and SslE expression following growth at 37°C was determined via western blot analysis (Fig 6A). As UTI89 already expresses a high level of SslE, mutation of *hns* did not lead to a detectable increase in protein expression by western blot analysis. Mutation of *fis*, on the other hand, significantly reduced SslE expression. In the UTI89*fis hns* double mutant, we detected a reduction in the level of SslE associated with the cell fraction (but not the supernatant fraction), suggesting that overall SslE expression is slightly reduced in this background compared to wild-type UTI89 (but increased compared to UTI89*fis*). Relative *sslE* transcript levels as determined via qRT-PCR showed a similar pattern, although the relative *sslE* transcript level detected in UTI89*fis hns* was similar to that of wild-type UTI89 (Fig 6B). Thus, in UTI89 at 37°C, Fis and H-NS play contrasting roles in SslE regulation; Fis strong activation and H-NS weak-moderate repression.

## Discussion

Since its identification as a vaccine candidate, the lipoprotein SslE has been characterized with respect to its function and immunogenicity. Immunization of mice with SslE provides protection in sepsis, intestinal and UTI models [20, 28], and SslE has also been identified as an immunogenic antigen following ETEC infection [31, 32]. SslE is secreted via a dedicated T2SS, but also remains partially attached to the cell surface [20, 21], thus explaining its role in the maturation of EPEC biofilms [21]. More recently, SslE has been shown to degrade mucin and this property is linked to the presence of a conserved M60-like zinc-metalloprotease domain [28–30, 85]. These findings, along with the high prevalence of the *sslE* gene in all *E*. *coli* pathotypes, suggest that SslE may contribute to long-term colonization of various mucosal sites [28, 29].

In this study, we observed that SslE expression is variable among different UPEC strains. The reference strains UTI89 and IHE3034 expressed and secreted high levels of SslE following growth in LB broth at 37°C. In contrast, the ST131 strain EC958 produced and secreted significantly less SslE under the same conditions. Further examination of UTI89 and EC958 revealed that at the transcriptional level, *sslE* is regulated by H-NS in a temperature-dependent manner, consistent with many other H-NS regulated genes in *E*. *coli* [86–89]. Both strains exhibited strong H-NS repression of *sslE* transcription at 22°C, a finding similar to that reported previously for ETEC H10407 [33]. At 37°C, however, strong transcription of *sslE* was observed in UTI89, and mutation of *hns* had only a minor effect on total SslE production. This finding was in contrast to that observed in EC958, where H-NS strongly repressed *sslE* expression at 37°C. These observations led us to investigate the genetic basis of *sslE* regulation at 37°C in UTI89 using transposon mutagenesis in combination with an *sslE* promoter-*lacZ*-reporter transcriptional fusion construct. Through this approach, we identified six genes associated with increased *sslE* promoter activity—*fis*, *typA*, *nusA*, *ygaZ*, *ygaH* and *dusB*. The *nusA* gene encodes a multi-functional transcription factor that plays an essential role in transcription elongation, pausing, termination and anti-termination [90–95]. Despite repeated attempts, we were unable to generate a specific *nusA* mutation in UTI89 and confirm its role in *sslE* regulation. We attribute this to the importance of *nusA* in viability [70, 96–98], and the observation that *nusA* is essential in *E*. *coli* K-12 [99, 100]. We note that a mutant containing a transposon insertion in the 3'-end of *nusA* (corresponding to residue 417 out of 495 amino acids) has been reported [101]. Our Tn5 insertions in *nusA* corresponded to nucleotide position 1131 and 1279 out of 1488 bp (amino acids 377 and 427), respectively, suggesting that mutations that disrupt the 3'-end of *nusA* are tolerated, while mutation of the entire *nusA* gene is lethal. It is also possible that the identification of *nusA* in our screen was due to its requirement for β-galactosidase synthesis as previously reported [102, 103]. However, the role of NusA in promoting factor-dependent transcription termination [104], together with the contribution of H-NS and StpA to this process [105], lends support to its role in *sslE* regulation, although this remains to be experimentally proven. One Tn*5* insertion mutant was also identified in each of the tandemly arranged hypothetical *ygaZ-ygaH* genes, respectively. However, we were unable to demonstrate a role for either gene in *sslE* regulation in subsequently constructed specific mutants, either in tandem or individually. The *dusB* gene is co-transcribed with *fis* [75], and therefore its identification is most likely linked to disruption of this operon. The *fis* and *typA* genes, which were identified in several independent Tn*5* mutants, were thus selected for study in greater detail.

We showed that the ribosomal binding GTPase TypA enhances SslE expression. Mutation of *typA* in UTI89 resulted in reduced SslE expression. This effect was only partially complemented by re-introduction of the *typA* gene, a result possibly linked to the use of a plasmid based system. TypA belongs to a ribosomal binding GTPase superfamily and is widely prevalent among different bacteria [78]. In *E*. *coli*, TypA contributes to the regulation of a range of virulence phenotypes, including flagella-mediated motility, resistance to antimicrobial peptides and the production of virulence determinants encoded by the locus of enterocyte effacement (LEE) pathogenicity island [67, 68, 106]; as well as K5 capsule production and growth at low temperatures [80, 107]. Our data adds SslE to the growing number of surface factors regulated by TypA in *E*. *coli*, although like many previous reports, the precise molecular mechanism by which TypA exerts its regulatory affect remains unclear [67, 68, 80]. TypA has not been shown to interact directly with DNA, with current evidence suggesting that TypA mediated regulation may be indirect and elicited at the translational level [68, 80–82, 108]. Further support of our results comes from a report that identified TypA as a regulator of the LEE-encoded type III secretion system in the EPEC strain E2348/69 [68]. Here, Grant *et al*. showed that in addition to controlling the LEE-encoded regulator Ler, TypA also regulates a ~170kDa secreted protein independent of the Type III secretion system. E2348/69 has been completely sequenced, and our analysis of its genome identified five genes that could encode proteins 160-180kDa in size: *sslE* (*yghJ*), *gltB* (encoding a glutamate synthase), *mukB* (encoding a chromosome partitioning protein), *yfaS* and *yfhM* (encoding hypothetical proteins). Of these, *sslE* is the only gene encoding a protein predicted to be secreted, suggesting that TypA also regulates SslE in E2348/69.

Several lines of evidence demonstrated a role for Fis in the regulation of *sslE* transcription. First, Tn*5* insertions were identified in both *fis* and *dusB*, which are co-transcribed as a single mRNA. Thus, independent Tn*5* insertions that either mutate *fis* or disrupt its transcription were identified. Second, mutation of *fis* in UTI89 led to a dramatic decrease in SslE expression and this could be complemented with a plasmid encoding the *fis* gene. Third, the mRNA transcript levels of *sslE* were reduced in a *fis* mutant and restored to wild-type level upon complementation. Fourth, cloning the *sslE* promoter region into a promoterless-*lacZ* reporter plasmid revealed that *sslE* promoter activity was approximately halved in a *fis* mutant background compared to wild-type. Finally, mutation of a putative consensus Fis-binding site in the *sslE* promoter region significantly reduced the activity of the *sslE* promoter. Fis is also known to activate the transcription of several other genes in *E*. *coli*, including the LEE transcriptional regulatory gene *ler* [66], and the enteroaggregative *E*. *coli* (EAEC) autotransporter toxin gene *pet* [65]. Fis also upregulates a wide variety of genes important during exponential growth including those involved in translation, metabolism, motility and nutrient transport [63]. Fis binding to DNA requires contact points to specific nucleotides, where explicit inclusion and exclusion rules at defined nucleotide positions necessary for Fis contact have been established [83, 84]. Our bioinformatic analysis identified two putative Fis-binding regions proximal to the *sslE* promoter that fulfilled these nucleotide inclusion/exclusion requirements. We further experimentally established that the putative F2 binding site, which is located immediately upstream of the -35 promoter element, is necessary for Fis activation of the *sslE* promoter. Disruption of F2 reduced the *sslE* promoter activity to a level similar to that observed in a *fis* mutant background. The F2 site consists of three overlapping Fis-binding consensus sequences [83, 84], and thus we chose to alter nucleotides that would disrupt all three binding sites simultaneously. The proximity of the F2 site to the *sslE* promoter suggests a mechanism typical of transcriptional activators: binding of Fis upstream of the *sslE* -35 promoter element and recruitment of RNA polymerase. Notably, our data does not rule out a role for the putative F1 binding site, as the function of upstream binding sites in Fis regulation can mediate subtle changes to local DNA topology [76]. Further experiments using purified Fis protein are now required to demonstrate its direct binding to the *sslE* promoter region.

The regulation of SslE by Fis was also demonstrated in strains IHE3034 and 536, and to a lesser extent in the low SslE-expressing strain EC958. A nucleotide alignment of the *sslE* promoter region from UTI89, IHE3034, 536 and EC958 revealed several nucleotide changes unique to both 536 and EC958 (S4 Fig). Notably, one of these changes occurs in the F2 Fis binding region, although the change is not in a key conserved nucleotide defined within the Fis-binding inclusion/exclusion rules. Furthermore, the higher level of SslE expression in 536 compared to EC958 suggests that this sequence change alone cannot explain the differences in SslE expression. Further analysis of the *sslE* promoter region in 87 completely sequenced *E*. *coli* genomes available on the NCBI database that were positive for *sslE* (S3 Table) revealed that there are no other nucleotide sequence changes in the F2 Fis binding sequence. In contrast to Fis, a stringent consensus DNA binding sequence for H-NS has not been defined, and it is generally accepted that H-NS binds to AT-rich and highly curved DNA [109–113]. Yang *et al*. previously reported that the ETEC H10407 *sslE* promoter region contains such features and binds specifically to purified H-NS protein [33], and we confirmed the AT-rich and curved DNA topology of the *sslE* promoter region in our strains using BEND-IT (http://hydra.icgeb.trieste.it/dna/bend_it.html). H-NS was not identified in our mutagenesis screen, which was designed to identify positive regulators of the *sslE* promoter. However, we showed that both Fis and H-NS alter the activity of the *sslE* promoter in a mutually antagonistic fashion. Fis and H-NS have been shown to co-regulate other genes in *E*. *coli*; for example *dps* and *nir* are repressed by the cooperative action of Fis and H-NS [114, 115], and both proteins also regulate transcription from the ribosomal RNA promoter (rrnB P1) as well as the *hns* promoter itself [116–118]. In *Shigella* and enteroinvasive *E*. *coli*, the *virF* gene is repressed by H-NS at 30°C and activated by Fis at 37°C [88]. In our experiments in UTI89, we showed that at 37°C mutation of *fis* leads to strong attenuation of SslE expression, and that this affect is partly overcome by the subsequent mutation of *hns*. This suggests an interplay between Fis and H-NS that may involve several mechanisms, including the direct competition for overlapping binding sites and temperature-mediated changes in DNA conformation that alter the binding affinity of both proteins. Both of these overlapping mechanisms could explain the molecular interactions that underpin Fis and H-NS regulation of *sslE* in UTI89.

SslE is a highly prevalent secreted and surface associated colonization factor of *E*. *coli*. In addition, SslE represents a promising vaccine antigen that provides broad protection from infection by multiple *E*. *coli* pathotypes. Overall, this work has identified differences in SslE expression by different UPEC strains at core body temperature, and provides evidence to demonstrate a role for Fis in the regulatory control of the *sslE* gene.

## Supporting Information

S1 Fig Differential expression of SslE among different clinical UPEC isolates. Western blot analysis of SslE using supernatant fractions from UTI89, UTI89*sslE* and 18 clinical UPEC isolates from our laboratory collection. SslE secretion varied among the different UPEC isolates.
(TIF)

S2 Fig Growth curves of UTI89 and EC958 and their respective *hns* mutants. Growth assays performed at 37°C under shaking conditions for (A) UTI89 and UTI89*hns*, (B) EC958 and EC958*hns*. In both strains, *hns* deletion mutants were attenuated in growth compared to their respective wild-type strains.
(TIF)

S3 Fig Effect of *hns* deletion on SslE expression by UTI89 and EC958 following growth at 37°C. Western blot analysis of SslE using preparations from (A) UTI89 and (B) EC958; each with their respective *hns* mutant and complemented strains. In each analysis, (i) whole-cell lysates and (iii) supernatant fractions were examined. In addition, (ii) a control for whole cell lysate samples was performed using an OmpA antibody.
(TIF)

S4 Fig Nucleotide alignment of the *sslE* promoter region between UTI89, IHE3034, 536 and EC968. The translation start site, transcription start site and promoter elements (-10 and -35 sequences) are indicated accordingly. The predicted Fis binding region (based on results from Fig 4) is boxed, with key nucleotide residues important for Fis binding indicated with a red overline. Nucleotide differences from UTI89 are highlighted.
(TIF)

S1 Table List of primers used in this study. (XLSX)

S2 Table Transposon mutant insertion site and β-galactosidase activity. Insertion site of the 13 Tn*5* mutants analyzed and the corresponding β-galactosidase activity for each mutant along with the control strains UTI89*lacI-Z sslE*::*lacZ* and UTI89*lacI-Z*.
(XLSX)

S3 Table List of complete *E*. *coli* genomes positive for *sslE* that were analyzed for nucleotide sequence variation in the putative F2 Fis-binding site of the *sslE* promoter region. The 87 completely sequenced *E*. *coli* strains are listed, along with associated isolate information, accession number and reference where available. All isolate information was derived from the NCBI database or corresponding reference.
(DOCX)
