# Supplementary figures and images for: Differential Regulation of the Surface-Exposed and Secreted SslE Lipoprotein in Extraintestinal Pathogenic Escherichia coli
Source: PLoS One. 2016 Sep 6;11(9):e0162391. doi: 10.1371/journal.pone.0162391 (PMC5012682; doi:10.1371/journal.pone.0162391)

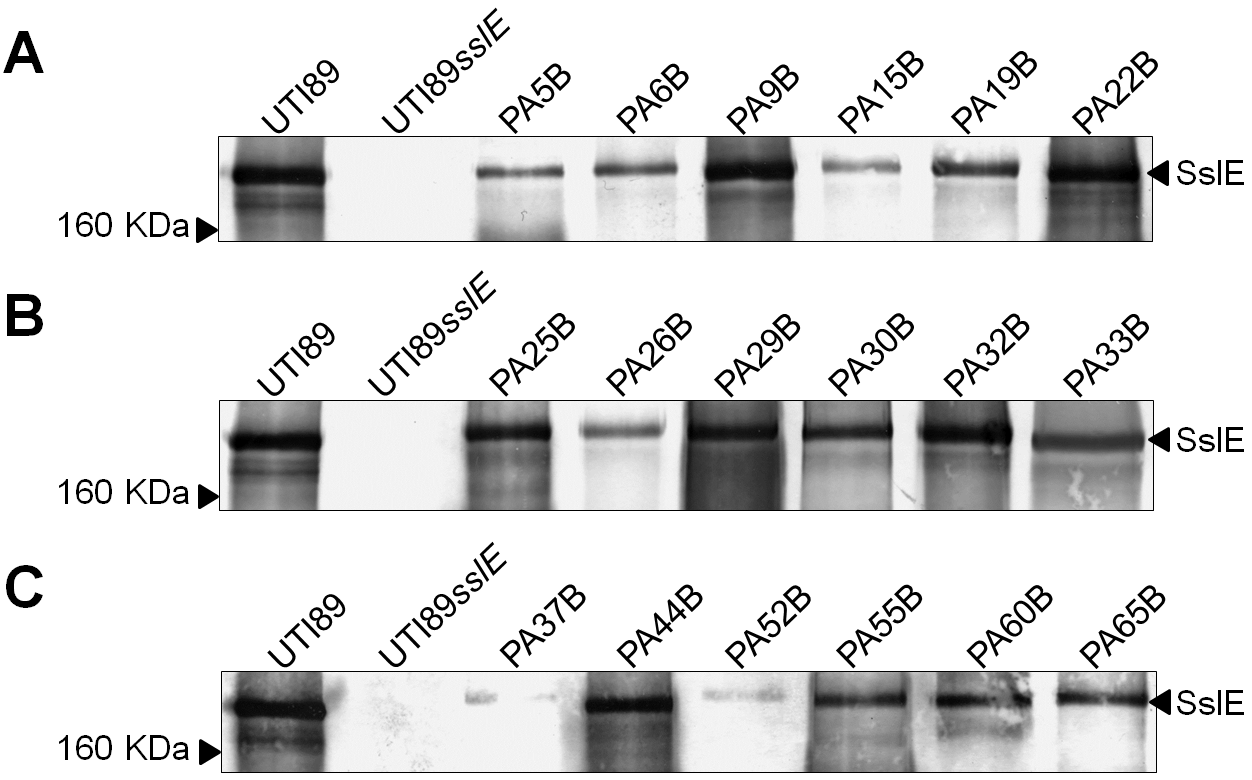

Supplement: S1 Fig — Western blot analysis of SslE using supernatant fractions from UTI89, UTI89sslE and 18 clinical UPEC isolates from our laboratory collection. SslE secretion varied among the different UPEC isolates. (TIF) [file pone.0162391.s001.tif]

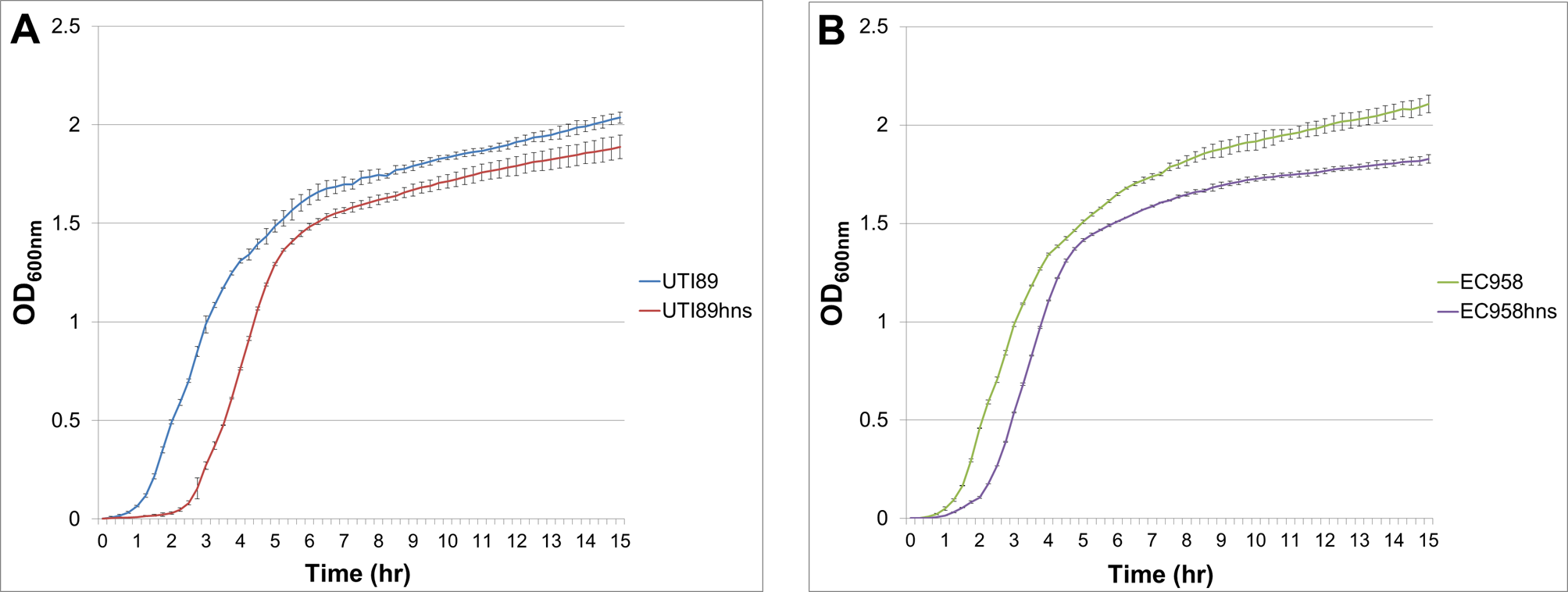

Supplement: S2 Fig — Growth assays performed at 37°C under shaking conditions for (A) UTI89 and UTI89hns, (B) EC958 and EC958hns. In both strains, hns deletion mutants were attenuated in growth compared to their respective wild-type strains. (TIF) [file pone.0162391.s002.tif]

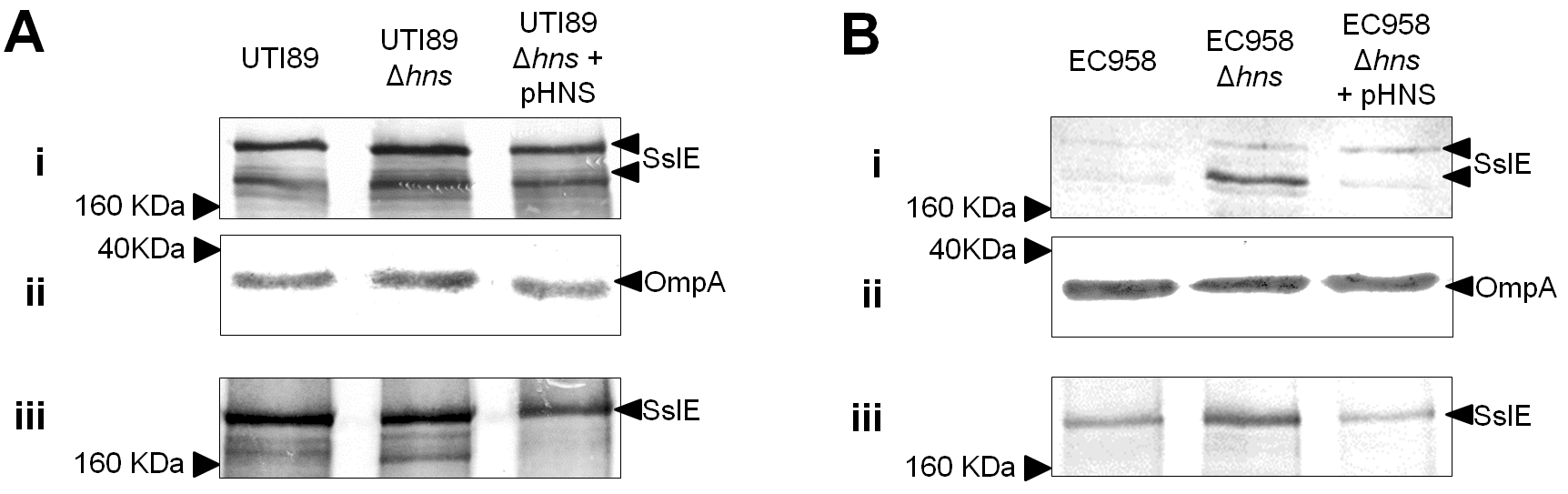

Supplement: S3 Fig — Western blot analysis of SslE using preparations from (A) UTI89 and (B) EC958; each with their respective hns mutant and complemented strains. In each analysis, (i) whole-cell lysates and (iii) supernatant fractions were examined. In addition, (ii) a control for whole cell lysate samples was performed using an OmpA antibody. (TIF) [file pone.0162391.s003.tif]

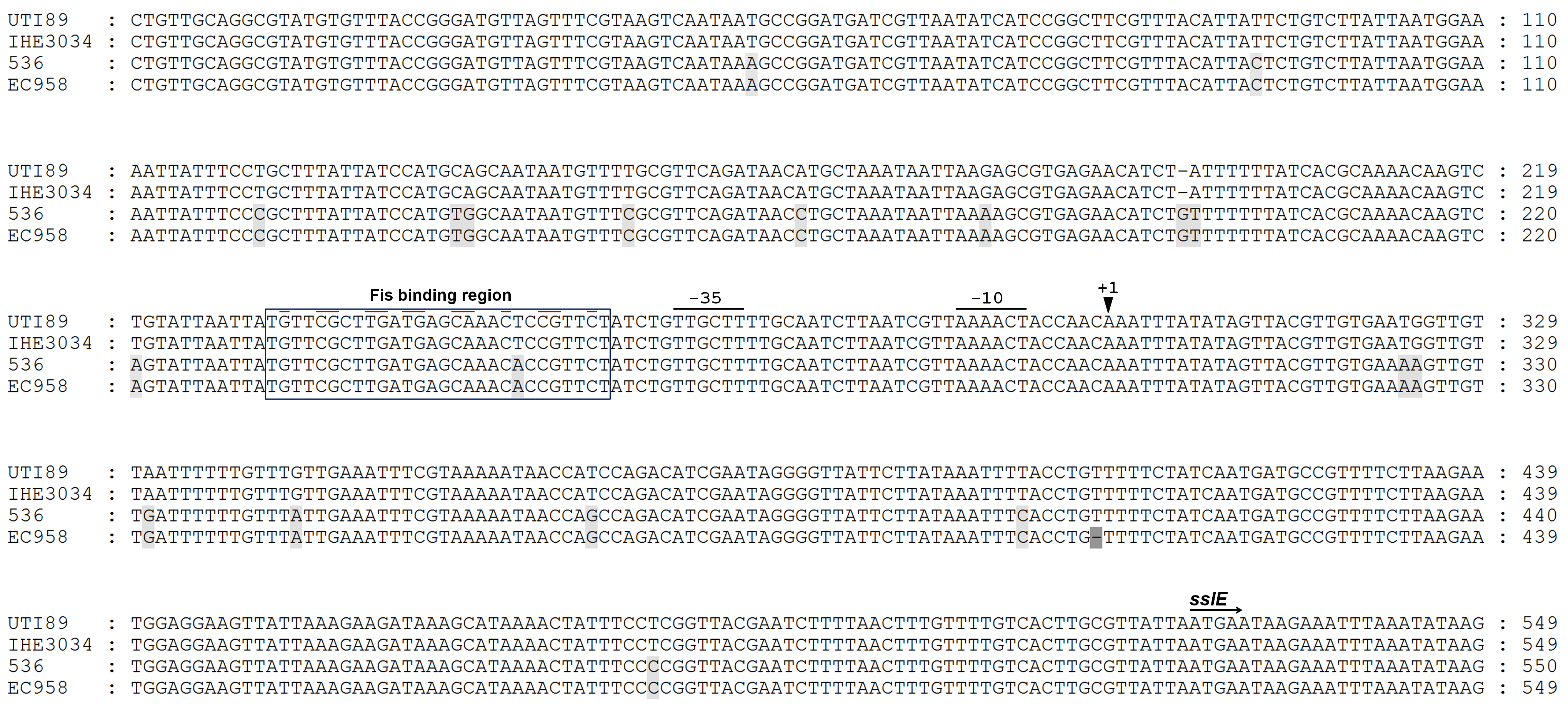

Supplement: S4 Fig — The translation start site, transcription start site and promoter elements (-10 and -35 sequences) are indicated accordingly. The predicted Fis binding region (based on results from Fig 4) is boxed, with key nucleotide residues important for Fis binding indicated with a red overline. Nucleotide differences from UTI89 are highlighted. (TIF) [file pone.0162391.s004.tif]
