# Supplementary material for: Differential Regulation of the Surface-Exposed and Secreted SslE Lipoprotein in Extraintestinal Pathogenic Escherichia coli
Source: PLoS One. 2016 Sep 6;11(9):e0162391. doi: 10.1371/journal.pone.0162391 (PMC5012682; doi:10.1371/journal.pone.0162391)
Supplement: S3 Table — The 87 completely sequenced E. coli strains are listed, along with associated isolate information, accession number and reference where available. All isolate information was derived from the NCBI database or corresponding reference. (DOCX) [file pone.0162391.s007.docx]

| **Strain** | **Isolate information (pathotype)** | **Accession number** | **Reference** |
| --- | --- | --- | --- |
| O1 | Turkey, colibacillosis (APEC) | CP000468.1 | [[1](#_ENREF_1)] |
| UTI89 | Human, cystitis (UPEC) | CP000243.1 | [[2](#_ENREF_2)] |
| PMV-1 | Murine peritonitis model strain | HG428755.1 | [[3](#_ENREF_3)] |
| SF-166 | Human, sepsis | CP012633.1 | [[4](#_ENREF_4)] |
| SF-088 | Human, sepsis | CP012635.1 | [[4](#_ENREF_4)] |
| SF-173 | Human, sepsis | CP012631.1 | [[4](#_ENREF_4)] |
| SF-468 | Human, sepsis | CP012625.1 | [[4](#_ENREF_4)] |
| UM146 | Human, Crohn’s disease (AIEC) | CP002167.1 | [[5](#_ENREF_5)] |
| IHE3034 | Human, neonatal meningitis (NMEC) | CP001969.1 | [[6](#_ENREF_6)] |
| RS218 | Human, neonatal meningitis (NMEC) | CP007149.1 | [[7](#_ENREF_7)] |
| S88 | Human, neonatal meningitis (NMEC) | CU928161.2 | [[8](#_ENREF_8)] |
| IMT5155 | Chicken, colibacillosis (APEC) | CP005930.1 | [[9](#_ENREF_9)] |
| LF82 | Human, Crohn’s disease (AIEC) | CU651637.1 | [[10](#_ENREF_10)] |
| NRG 857C | Human, Crohn’s disease (AIEC) | CP001855.1 | [[11](#_ENREF_11)] |
| ED1a | Healthy human stool | CU928162.2 | [[12](#_ENREF_12)] |
| SE15 | Healthy human stool | AP009378.1 | [[13](#_ENREF_13)] |
| ST648 | Human, pleural effusion | CP008697.1 | - |
| ST2747 | Human stool isolate from patient with UTI | CP007392.1 | [[14](#_ENREF_14)] |
| ST540 | Human stool isolate from patient with UTI | CP007265.1 | [[14](#_ENREF_14)] |
| VR50 | Human, asymptomatic bacteriuria | CP011134.1 | [[15](#_ENREF_15)] |
| IAI39 | Human, pyelonephritis (UPEC) | CU928164.2 | [[12](#_ENREF_12)] |
| 042 | Human, diarrhoea (EAEC) | FN554766.1 | [[16](#_ENREF_16)] |
| CE10 | Human, neonatal meningitis | CP003034.1 | [[17](#_ENREF_17)] |
| 536 | Human, pyelonephritis (UPEC) | CP000247.1 | [[18](#_ENREF_18)] |
| ECONIH1 | Perirectal isolate from stem cell transplant patient | CP009859.1 | [[19](#_ENREF_19)] |
| JJ1886 | Human, urosepsis (UPEC) | CP006784.1 | [[20](#_ENREF_20)] |
| 83972 | Human, asymptomatic bacteriuria | CP001671.1 | [[21](#_ENREF_21)] |
| clone D i2 | Human, cystitis (UPEC) | CP002211.1 | [[22](#_ENREF_22)] |
| clone D i14 | Human, cystitis (UPEC) | CP002212.1 | [[22](#_ENREF_22)] |
| EC958 | Human, cystitis (UPEC) | HG941718.1 | [[23](#_ENREF_23)] |
| uk_P46212 | Human, cystitis (UPEC) | CP013658.1 | [[24](#_ENREF_24)] |
| Nissle 1917 | Probiotic strain | CP007799.1 | [[25](#_ENREF_25)] |
| 2009C-3133 | Human, diarrhoea (STEC) | CP013025.1 | [[26](#_ENREF_26)] |
| Santai | O157:H16 EHEC strain from rectum of a healthy duck | CP007592.1 | - |
| RV308 | *E. coli* K-12 (ATCC 31608) | LM995446.1 | - |
| HMS174 | *E. coli* K-12 (ATCC 47011) | LM993812.1 | - |
| GM4792 | *E. coli* K-12 | CP011343.2 | [[27](#_ENREF_27)] |
| 1303 | Cow, acute bovine mastitis | CP009166.1 | [[28](#_ENREF_28)] |
| DH10β | *E. coli* K-12 | CP000948.1 | [[29](#_ENREF_29)] |
| SMS-3-5 | Metal-contaminated industrial coastal environment isolate | CP000970.1 | [[30](#_ENREF_30)] |
| ER2796 | *E. coli* K-12 | CP009644.1 | [[31](#_ENREF_31)] |
| FAP1 | ESBL producing *E. coli* from pig faeces | CP009578.1 | [[32](#_ENREF_32)] |
| BW25113 | *E. coli* K-12 | CP009273.1 | [[33](#_ENREF_33)] |
| KLY | *E. coli* K-12 | CP008801.1 | [[34](#_ENREF_34)] |
| MG1655 | *E. coli* K-12 | CP009685.1 | [[35](#_ENREF_35)] |
| MC4100 | *E. coli* K-12 | HG738867.1 | [[36](#_ENREF_36)] |
| NCM3722 | *E. coli* K-12 | CP011495.1 | [[37](#_ENREF_37)] |
| UMNF18 | Porcine ETEC | AGTD01000001.1 | [[38](#_ENREF_38)] |
| RR1 | *E. coli* K-12 (KCTC:2134) | CP011113.1 | - |
| W3110 | *E. coli* K-12 | AP009048.1 | [[39](#_ENREF_39)] |
| DH1 | *E. coli* K-12 | AP012030.1 | [[40](#_ENREF_40)] |
| P12b | H17 flagella reference strain | CP002291.1 | [[41](#_ENREF_41)] |
| UMNK88 | Porcine ETEC | CP002729.1 | [[38](#_ENREF_38)] |
| IAI1 | Healthy human stool | CU928160.2 | [[12](#_ENREF_12)] |
| SEC470 | Piglet, diarrhoea | CP007594.1 | - |
| H10407 | Human, diarrhoea (ETEC) | FN649414.1 | [[42](#_ENREF_42)] |
| CB9615 | Human, diarrhoea (atypical EPEC) | CP001846.1 | [[43](#_ENREF_43)] |
| RM12579 | Human, diarrhoea (atypical EPEC) | CP003109.1 | [[44](#_ENREF_44)] |
| 94-3024 | Human, hemorrhagic colitis (STEC) | CP009106.2 | [[45](#_ENREF_45)] |
| 789 | Poultry, colisepticaemia (APEC) | CP010315.1 | [[46](#_ENREF_46)] |
| REL606 | *E. coli* B | CP000819.1 | [[47](#_ENREF_47)] |
| BL21 (DE3) | *E. coli* B | CP001509.3 | [[47](#_ENREF_47)] |
| YD786 | Human, UTI (UPEC) | CP013112.1 | - |
| 2012C-4227 | Human, diarrhoea (STEC) | CP013029.1 | [[26](#_ENREF_26)] |
| W | *E. coli* W (ATCC 9637) | CP002185.1 | [[48](#_ENREF_48)] |
| LY180 | *E. coli* W derivative | CP006584.1 | [[49](#_ENREF_49)] |
| KO11FL | *E. coli* W derivative | CP002970.1 | [[50](#_ENREF_50)] |
| 55989 | Human, gastroenteritis (EAEC) | CU928145.2 | - |
| RS76 | Chicken, cellulitis (APEC) | CP013048.1 | [[51](#_ENREF_51)] |
| CI5 | Human, pyelonephritis (UPEC) | CP011018.1 | [[52](#_ENREF_52)] |
| 11128 | Human, bloody diarrhoea (EHEC) | AP010960.1 | [[53](#_ENREF_53)] |
| 12009 | Human, bloody diarrhoea (EHEC) | AP010958.1 | [[53](#_ENREF_53)] |
| CFSAN029787 | Human, diarrhoea (EIEC) | CP011416.1 | [[54](#_ENREF_54)] |
| C227-11 | Human, bloody diarrhoea (EAEC) | CP011331.1 | [[55](#_ENREF_55)] |
| ECC-1470 | Cow, persistent bovine mastitis | CP010344.1 | [[28](#_ENREF_28)] |
| HUSEC2011 | Human, haemolytic uremic syndrome (EHEC) | HF572917.2 | - |
| RM9387 | Cattle faeces (STEC) | CP009104.1 | [[45](#_ENREF_45)] |
| 2009EL-2050 | Human, bloody diarrhoea (EAHEC) | CP003297.1 | [[56](#_ENREF_56)] |
| 2009EL-2071 | Human, bloody diarrhoea (EAHEC) | CP003301.1 | [[56](#_ENREF_56)] |
| 2011C-3493 | Human, bloody diarrhoea (EAHEC) | CP003289.1 | [[56](#_ENREF_56)] |
| E24377A | Human, diarrhoea (ETEC) | CP000800.1 | [[57](#_ENREF_57)] |
| B7A | Human, diarrhoea (ETEC) | CP005998.1 | [[57](#_ENREF_57)] |
| SE11 | Healthy human stool | AP009240.1 | [[58](#_ENREF_58)] |
| E2348/69 | Human, diarrhoea (EPEC) | FM180568.1 | [[59](#_ENREF_59)] |
| PCN033 | Pig, meningitis (ExPEC) | CP006632.1 | [[60](#_ENREF_60)] |
| UMN026 | Human, cystitis (UPEC) | CU928163.2 | [[12](#_ENREF_12)] |
| HS | Healthy human stool | CP000802.1 | [[57](#_ENREF_57)] |

1. Johnson TJ, Kariyawasam S, Wannemuehler Y, Mangiamele P, Johnson SJ, Doetkott C, et al. The genome sequence of avian pathogenic *Escherichia coli* strain O1:K1:H7 shares strong similarities with human extraintestinal pathogenic *E. coli* genomes. J Bacteriol. 2007;189(8):3228-36. Epub 2007/02/13. doi: 10.1128/jb.01726-06. PubMed PMID: 17293413; PubMed Central PMCID: PMC1855855.

2. Chen SL, Hung CS, Xu J, Reigstad CS, Magrini V, Sabo A, et al. Identification of genes subject to positive selection in uropathogenic strains of *Escherichia coli*: a comparative genomics approach. Proc Natl Acad Sci U S A. 2006;103(15):5977-82. Epub 2006/04/06. doi: 10.1073/pnas.0600938103. PubMed PMID: 16585510; PubMed Central PMCID: PMC1424661.

3. Peris-Bondia F, Muraille E, Van Melderen L. Complete Genome Sequence of the *Escherichia coli* PMV-1 Strain, a Model Extraintestinal Pathogenic *E. coliE. coli* Strain Used for Host-Pathogen Interaction Studies. Genome Announc. 2013;1(5). Epub 2013/10/26. doi: 10.1128/genomeA.00913-13. PubMed PMID: 24158560; PubMed Central PMCID: PMC3813190.

4. Stephens CM, Skerker JM, Sekhon MS, Arkin AP, Riley LW. Complete Genome Sequences of Four *Escherichia coli* ST95 Isolates from Bloodstream Infections. Genome Announc. 2015;3(6). Epub 2015/11/07. doi: 10.1128/genomeA.01241-15. PubMed PMID: 26543109; PubMed Central PMCID: PMC4645194.

5. Krause DO, Little AC, Dowd SE, Bernstein CN. Complete genome sequence of adherent invasive *Escherichia coli* UM146 isolated from Ileal Crohn's disease biopsy tissue. J Bacteriol. 2011;193(2):583. Epub 2010/11/16. doi: 10.1128/jb.01290-10. PubMed PMID: 21075930; PubMed Central PMCID: PMC3019814.

6. Moriel DG, Bertoldi I, Spagnuolo A, Marchi S, Rosini R, Nesta B, et al. Identification of protective and broadly conserved vaccine antigens from the genome of extraintestinal pathogenic *Escherichia coli*. Proc Natl Acad Sci U S A. 2010;107(20):9072-7. Epub 2010/05/05. doi: 10.1073/pnas.0915077107. PubMed PMID: 20439758; PubMed Central PMCID: PMC2889118.

7. Wijetunge DS, Katani R, Kapur V, Kariyawasam S. Complete Genome Sequence of *Escherichia coli* Strain RS218 (O18:H7:K1), Associated with Neonatal Meningitis. Genome Announc. 2015;3(4). Epub 2015/07/25. doi: 10.1128/genomeA.00804-15. PubMed PMID: 26205862; PubMed Central PMCID: PMC4513156.

8. Peigne C, Bidet P, Mahjoub-Messai F, Plainvert C, Barbe V, Medigue C, et al. The plasmid of *Escherichia coli* strain S88 (O45:K1:H7) that causes neonatal meningitis is closely related to avian pathogenic *E. coliE. coli* plasmids and is associated with high-level bacteremia in a neonatal rat meningitis model. Infect Immun. 2009;77(6):2272-84. Epub 2009/03/25. doi: 10.1128/IAI.01333-08. PubMed PMID: 19307211; PubMed Central PMCID: PMC2687354.

9. Zhu Ge X, Jiang J, Pan Z, Hu L, Wang S, Wang H, et al. Comparative genomic analysis shows that avian pathogenic *Escherichia coli* isolate IMT5155 (O2:K1:H5; ST complex 95, ST140) shares close relationship with ST95 APEC O1:K1 and human ExPEC O18:K1 strains. PLoS One. 2014;9(11):e112048. Epub 2014/11/15. doi: 10.1371/journal.pone.0112048. PubMed PMID: 25397580; PubMed Central PMCID: PMC4232414.

10. Miquel S, Peyretaillade E, Claret L, de Vallee A, Dossat C, Vacherie B, et al. Complete genome sequence of Crohn's disease-associated adherent-invasive *E. coliE. coli* strain LF82. PLoS One. 2010;5(9). Epub 2010/09/24. doi: 10.1371/journal.pone.0012714. PubMed PMID: 20862302; PubMed Central PMCID: PMC2941450.

11. Nash JH, Villegas A, Kropinski AM, Aguilar-Valenzuela R, Konczy P, Mascarenhas M, et al. Genome sequence of adherent-invasive *Escherichia coli* and comparative genomic analysis with other *E. coli* pathotypes. BMC Genomics. 2010;11:667. Epub 2010/11/27. doi: 10.1186/1471-2164-11-667. PubMed PMID: 21108814; PubMed Central PMCID: PMC3091784.

12. Touchon M, Hoede C, Tenaillon O, Barbe V, Baeriswyl S, Bidet P, et al. Organised genome dynamics in the *Escherichia coli* species results in highly diverse adaptive paths. PLoS Genet. 2009;5(1):e1000344. Epub 2009/01/24. doi: 10.1371/journal.pgen.1000344. PubMed PMID: 19165319; PubMed Central PMCID: PMC2617782.

13. Toh H, Oshima K, Toyoda A, Ogura Y, Ooka T, Sasamoto H, et al. Complete genome sequence of the wild-type commensal *Escherichia coli* strain SE15, belonging to phylogenetic group B2. J Bacteriol. 2010;192(4):1165-6. Epub 2009/12/17. doi: 10.1128/jb.01543-09. PubMed PMID: 20008064; PubMed Central PMCID: PMC2812965.

14. Xavier BB, Vervoort J, Stewardson A, Adriaenssens N, Coenen S, Harbarth S, et al. Complete Genome Sequences of Nitrofurantoin-Sensitive and -Resistant *Escherichia coli* ST540 and ST2747 Strains. Genome Announc. 2014;2(2). Epub 2014/04/12. doi: 10.1128/genomeA.00239-14. PubMed PMID: 24723707; PubMed Central PMCID: PMC3983296.

15. Beatson SA, Ben Zakour NL, Totsika M, Forde BM, Watts RE, Mabbett AN, et al. Molecular analysis of asymptomatic bacteriuria *Escherichia coli* strain VR50 reveals adaptation to the urinary tract by gene acquisition. Infect Immun. 2015;83(5):1749-64. Epub 2015/02/11. doi: 10.1128/iai.02810-14. PubMed PMID: 25667270; PubMed Central PMCID: PMC4399054.

16. Chaudhuri RR, Sebaihia M, Hobman JL, Webber MA, Leyton DL, Goldberg MD, et al. Complete genome sequence and comparative metabolic profiling of the prototypical enteroaggregative *Escherichia coli* strain 042. PLoS One. 2010;5(1):e8801. Epub 2010/01/26. doi: 10.1371/journal.pone.0008801. PubMed PMID: 20098708; PubMed Central PMCID: PMC2808357.

17. Lu S, Zhang X, Zhu Y, Kim KS, Yang J, Jin Q. Complete genome sequence of the neonatal-meningitis-associated *Escherichia coli* strain CE10. J Bacteriol. 2011;193(24):7005. Epub 2011/11/30. doi: 10.1128/jb.06284-11. PubMed PMID: 22123760; PubMed Central PMCID: PMC3232859.

18. Hochhut B, Wilde C, Balling G, Middendorf B, Dobrindt U, Brzuszkiewicz E, et al. Role of pathogenicity island-associated integrases in the genome plasticity of uropathogenic *Escherichia coli* strain 536. Mol Microbiol. 2006;61(3):584-95. Epub 2006/08/02. doi: 10.1111/j.1365-2958.2006.05255.x. PubMed PMID: 16879640.

19. Conlan S, Thomas PJ, Deming C, Park M, Lau AF, Dekker JP, et al. Single-molecule sequencing to track plasmid diversity of hospital-associated carbapenemase-producing *Enterobacteriaceae*. Sci Transl Med. 2014;6(254):254ra126. Epub 2014/09/19. doi: 10.1126/scitranslmed.3009845. PubMed PMID: 25232178; PubMed Central PMCID: PMC4203314.

20. Andersen PS, Stegger M, Aziz M, Contente-Cuomo T, Gibbons HS, Keim P, et al. Complete Genome Sequence of the Epidemic and Highly Virulent CTX-M-15-Producing H30-Rx Subclone of *Escherichia coli* ST131. Genome Announc. 2013;1(6). Epub 2013/12/07. doi: 10.1128/genomeA.00988-13. PubMed PMID: 24309736; PubMed Central PMCID: PMC3853059.

21. Zdziarski J, Brzuszkiewicz E, Wullt B, Liesegang H, Biran D, Voigt B, et al. Host imprints on bacterial genomes—rapid, divergent evolution in individual patients. PLoS Pathog. 2010;6(8):e1001078. Epub 2010/09/25. doi: 10.1371/journal.ppat.1001078. PubMed PMID: 20865122; PubMed Central PMCID: PMC2928814.

22. Reeves PR, Liu B, Zhou Z, Li D, Guo D, Ren Y, et al. Rates of mutation and host transmission for an *Escherichia coli* clone over 3 years. PLoS One. 2011;6(10):e26907. Epub 2011/11/03. doi: 10.1371/journal.pone.0026907. PubMed PMID: 22046404; PubMed Central PMCID: PMC3203180.

23. Forde BM, Ben Zakour NL, Stanton-Cook M, Phan MD, Totsika M, Peters KM, et al. The complete genome sequence of *Escherichia coli* EC958: a high quality reference sequence for the globally disseminated multidrug resistant *E. coli* O25b:H4-ST131 clone. PLoS One. 2014;9(8):e104400. Epub 2014/08/16. doi: 10.1371/journal.pone.0104400. PubMed PMID: 25126841; PubMed Central PMCID: PMC4134206.

24. Stoesser N, Sheppard A, Pankhurst L, de Maio N, Moore CE, Sebra R, et al. Evolutionary history of the global emergence of the *Escherichia coli* epidemic clone ST131. bioRxiv. 2015. doi: 10.1101/030668.

25. Reister M, Hoffmeier K, Krezdorn N, Rotter B, Liang C, Rund S, et al. Complete genome sequence of the gram-negative probiotic *Escherichia coli* strain Nissle 1917. J Biotechnol. 2014;187:106-7. Epub 2014/08/06. doi: 10.1016/j.jbiotec.2014.07.442. PubMed PMID: 25093936.

26. Lindsey RL, Knipe K, Rowe L, Garcia-Toledo L, Loparev V, Juieng P, et al. Complete Genome Sequences of Two Shiga Toxin-Producing *Escherichia coli* Strains from Serotypes O119:H4 and O165:H25. Genome Announc. 2015;3(6). Epub 2015/12/19. doi: 10.1128/genomeA.01496-15. PubMed PMID: 26679598; PubMed Central PMCID: PMC4683243.

27. Zhang YC, Zhang Y, Zhu BR, Zhang BW, Ni C, Zhang DY, et al. Genome sequences of two closely related strains of *Escherichia coli* K-12 GM4792. Stand Genomic Sci. 2015;10:125. Epub 2015/12/15. doi: 10.1186/s40793-015-0114-x. PubMed PMID: 26664654; PubMed Central PMCID: PMC4675052.

28. Leimbach A, Poehlein A, Witten A, Scheutz F, Schukken Y, Daniel R, et al. Complete Genome Sequences of *Escherichia coli* Strains 1303 and ECC-1470 Isolated from Bovine Mastitis. Genome Announc. 2015;3(2). Epub 2015/03/31. doi: 10.1128/genomeA.00182-15. PubMed PMID: 25814601; PubMed Central PMCID: PMC4384141.

29. Durfee T, Nelson R, Baldwin S, Plunkett G, 3rd, Burland V, Mau B, et al. The complete genome sequence of *Escherichia coli* DH10β: insights into the biology of a laboratory workhorse. J Bacteriol. 2008;190(7):2597-606. Epub 2008/02/05. doi: 10.1128/jb.01695-07. PubMed PMID: 18245285; PubMed Central PMCID: PMC2293198.

30. Fricke WF, Wright MS, Lindell AH, Harkins DM, Baker-Austin C, Ravel J, et al. Insights into the environmental resistance gene pool from the genome sequence of the multidrug-resistant environmental isolate *Escherichia coli* SMS-3-5. J Bacteriol. 2008;190(20):6779-94. Epub 2008/08/19. doi: 10.1128/jb.00661-08. PubMed PMID: 18708504; PubMed Central PMCID: PMC2566207.

31. Anton BP, Mongodin EF, Agrawal S, Fomenkov A, Byrd DR, Roberts RJ, et al. Complete Genome Sequence of ER2796, a DNA Methyltransferase-Deficient Strain of *Escherichia coli* K-12. PLoS One. 2015;10(5):e0127446. Epub 2015/05/27. doi: 10.1371/journal.pone.0127446. PubMed PMID: 26010885; PubMed Central PMCID: PMC4444293.

32. de Been M, Lanza VF, de Toro M, Scharringa J, Dohmen W, Du Y, et al. Dissemination of cephalosporin resistance genes between *Escherichia coli* strains from farm animals and humans by specific plasmid lineages. PLoS Genet. 2014;10(12):e1004776. Epub 2014/12/19. doi: 10.1371/journal.pgen.1004776. PubMed PMID: 25522320; PubMed Central PMCID: PMC4270446.

33. Grenier F, Matteau D, Baby V, Rodrigue S. Complete Genome Sequence of *Escherichia coli* BW25113. Genome Announc. 2014;2(5). Epub 2014/10/18. doi: 10.1128/genomeA.01038-14. PubMed PMID: 25323716; PubMed Central PMCID: PMC4200154.

34. Fridman O, Goldberg A, Ronin I, Shoresh N, Balaban NQ. Optimization of lag time underlies antibiotic tolerance in evolved bacterial populations. Nature. 2014;513(7518):418-21. Epub 2014/07/22. doi: 10.1038/nature13469. PubMed PMID: 25043002.

35. Berlin K, Koren S, Chin CS, Drake JP, Landolin JM, Phillippy AM. Assembling large genomes with single-molecule sequencing and locality-sensitive hashing. Nat Biotechnol. 2015;33(6):623-30. Epub 2015/05/26. doi: 10.1038/nbt.3238. PubMed PMID: 26006009.

36. Laehnemann D, Pena-Miller R, Rosenstiel P, Beardmore R, Jansen G, Schulenburg H. Genomics of rapid adaptation to antibiotics: convergent evolution and scalable sequence amplification. Genome Biol Evol. 2014;6(6):1287-301. Epub 2014/05/23. doi: 10.1093/gbe/evu106. PubMed PMID: 24850796; PubMed Central PMCID: PMC4079197.

37. Brown SD, Jun S. Complete Genome Sequence of *Escherichia coli* NCM3722. Genome Announc. 2015;3(4). Epub 2015/08/08. doi: 10.1128/genomeA.00879-15. PubMed PMID: 26251500; PubMed Central PMCID: PMC4541272.

38. Shepard SM, Danzeisen JL, Isaacson RE, Seemann T, Achtman M, Johnson TJ. Genome sequences and phylogenetic analysis of K88- and F18-positive porcine enterotoxigenic *Escherichia coli*. J Bacteriol. 2012;194(2):395-405. Epub 2011/11/15. doi: 10.1128/jb.06225-11. PubMed PMID: 22081385; PubMed Central PMCID: PMC3256668.

39. Hayashi K, Morooka N, Yamamoto Y, Fujita K, Isono K, Choi S, et al. Highly accurate genome sequences of *Escherichia coli* K-12 strains MG1655 and W3110. Mol Syst Biol. 2006;2:2006 0007. Epub 2006/06/02. doi: 10.1038/msb4100049. PubMed PMID: 16738553; PubMed Central PMCID: PMC1681481.

40. Suzuki S, Ono N, Furusawa C, Ying BW, Yomo T. Comparison of sequence reads obtained from three next-generation sequencing platforms. PLoS One. 2011;6(5):e19534. Epub 2011/05/26. doi: 10.1371/journal.pone.0019534. PubMed PMID: 21611185; PubMed Central PMCID: PMC3096631.

41. Liu B, Hu B, Zhou Z, Guo D, Guo X, Ding P, et al. A novel non-homologous recombination-mediated mechanism for *Escherichia coli* unilateral flagellar phase variation. Nucleic Acids Res. 2012;40(10):4530-8. Epub 2012/01/31. doi: 10.1093/nar/gks040. PubMed PMID: 22287625; PubMed Central PMCID: PMC3378880.

42. Crossman LC, Chaudhuri RR, Beatson SA, Wells TJ, Desvaux M, Cunningham AF, et al. A commensal gone bad: complete genome sequence of the prototypical enterotoxigenic *Escherichia coli* strain H10407. J Bacteriol. 2010;192(21):5822-31. Epub 2010/08/31. doi: 10.1128/jb.00710-10. PubMed PMID: 20802035; PubMed Central PMCID: PMC2953697.

43. Zhou Z, Li X, Liu B, Beutin L, Xu J, Ren Y, et al. Derivation of *Escherichia coli* O157:H7 from its O55:H7 precursor. PLoS One. 2010;5(1):e8700. Epub 2010/01/22. doi: 10.1371/journal.pone.0008700. PubMed PMID: 20090843; PubMed Central PMCID: PMC2806823.

44. Kyle JL, Cummings CA, Parker CT, Quinones B, Vatta P, Newton E, et al. *Escherichia coli* serotype O55:H7 diversity supports parallel acquisition of bacteriophage at Shiga toxin phage insertion sites during evolution of the O157:H7 lineage. J Bacteriol. 2012;194(8):1885-96. Epub 2012/02/14. doi: 10.1128/jb.00120-12. PubMed PMID: 22328665; PubMed Central PMCID: PMC3318487.

45. Yan X, Fratamico PM, Bono JL, Baranzoni GM, Chen CY. Genome sequencing and comparative genomics provides insights on the evolutionary dynamics and pathogenic potential of different H-serotypes of Shiga toxin-producing *Escherichia coli* O104. BMC Microbiol. 2015;15:83. Epub 2015/04/19. doi: 10.1186/s12866-015-0413-9. PubMed PMID: 25887577; PubMed Central PMCID: PMC4393859.

46. Huja S, Oren Y, Trost E, Brzuszkiewicz E, Biran D, Blom J, et al. Genomic avenue to avian colisepticemia. MBio. 2015;6(1). Epub 2015/01/15. doi: 10.1128/mBio.01681-14. PubMed PMID: 25587010; PubMed Central PMCID: PMC4313913.

47. Jeong H, Barbe V, Lee CH, Vallenet D, Yu DS, Choi SH, et al. Genome sequences of *Escherichia coli* B strains REL606 and BL21(DE3). J Mol Biol. 2009;394(4):644-52. Epub 2009/09/30. doi: 10.1016/j.jmb.2009.09.052. PubMed PMID: 19786035.

48. Archer CT, Kim JF, Jeong H, Park JH, Vickers CE, Lee SY, et al. The genome sequence of *E. coli* W (ATCC 9637): comparative genome analysis and an improved genome-scale reconstruction of *E. coli*. BMC Genomics. 2011;12:9. Epub 2011/01/07. doi: 10.1186/1471-2164-12-9. PubMed PMID: 21208457; PubMed Central PMCID: PMC3032704.

49. Geddes RD, Wang X, Yomano LP, Miller EN, Zheng H, Shanmugam KT, et al. Polyamine transporters and polyamines increase furfural tolerance during xylose fermentation with ethanologenic *Escherichia coli* strain LY180. Appl Environ Microbiol. 2014;80(19):5955-64. Epub 2014/07/27. doi: 10.1128/aem.01913-14. PubMed PMID: 25063650; PubMed Central PMCID: PMC4178697.

50. Turner PC, Yomano LP, Jarboe LR, York SW, Baggett CL, Moritz BE, et al. Optical mapping and sequencing of the *Escherichia coli* KO11 genome reveal extensive chromosomal rearrangements, and multiple tandem copies of the *Zymomonas mobilis* *pdc* and *adhB* genes. J Ind Microbiol Biotechnol. 2012;39(4):629-39. Epub 2011/11/15. doi: 10.1007/s10295-011-1052-2. PubMed PMID: 22075923.

51. Maluta RP, Nicholson B, Logue CM, Nolan LK, Rojas TC, Dias da Silveira W. Complete Genomic Sequence of an Avian Pathogenic *Escherichia coli* Strain of Serotype O7:HNT. Genome Announc. 2016;4(1). Epub 2016/01/30. doi: 10.1128/genomeA.01611-15. PubMed PMID: 26823578.

52. Mehershahi KS, Abraham SN, Chen SL. Complete Genome Sequence of Uropathogenic *Escherichia coli* Strain CI5. Genome Announc. 2015;3(3). Epub 2015/05/30. doi: 10.1128/genomeA.00558-15. PubMed PMID: 26021932; PubMed Central PMCID: PMC4447917.

53. Ogura Y, Ooka T, Iguchi A, Toh H, Asadulghani M, Oshima K, et al. Comparative genomics reveal the mechanism of the parallel evolution of O157 and non-O157 enterohemorrhagic *Escherichia coli*. Proc Natl Acad Sci U S A. 2009;106(42):17939-44. Epub 2009/10/10. doi: 10.1073/pnas.0903585106. PubMed PMID: 19815525; PubMed Central PMCID: PMC2764950.

54. Pettengill EA, Hoffmann M, Binet R, Roberts RJ, Payne J, Allard M, et al. Complete Genome Sequence of Enteroinvasive *Escherichia coli* O96:H19 Associated with a Severe Foodborne Outbreak. Genome Announc. 2015;3(4). Epub 2015/08/08. doi: 10.1128/genomeA.00883-15. PubMed PMID: 26251502; PubMed Central PMCID: PMC4541276.

55. Beaulaurier J, Zhang XS, Zhu S, Sebra R, Rosenbluh C, Deikus G, et al. Single molecule-level detection and long read-based phasing of epigenetic variations in bacterial methylomes. Nat Commun. 2015;6:7438. Epub 2015/06/16. doi: 10.1038/ncomms8438. PubMed PMID: 26074426; PubMed Central PMCID: PMC4490391.

56. Ahmed SA, Awosika J, Baldwin C, Bishop-Lilly KA, Biswas B, Broomall S, et al. Genomic comparison of *Escherichia coli* O104:H4 isolates from 2009 and 2011 reveals plasmid, and prophage heterogeneity, including shiga toxin encoding phage stx2. PLoS One. 2012;7(11):e48228. Epub 2012/11/08. doi: 10.1371/journal.pone.0048228. PubMed PMID: 23133618; PubMed Central PMCID: PMC3486847.

57. Rasko DA, Rosovitz MJ, Myers GS, Mongodin EF, Fricke WF, Gajer P, et al. The pangenome structure of *Escherichia coli*: comparative genomic analysis of *E. coli* commensal and pathogenic isolates. J Bacteriol. 2008;190(20):6881-93. Epub 2008/08/05. doi: 10.1128/jb.00619-08. PubMed PMID: 18676672; PubMed Central PMCID: PMC2566221.

58. Oshima K, Toh H, Ogura Y, Sasamoto H, Morita H, Park SH, et al. Complete genome sequence and comparative analysis of the wild-type commensal *Escherichia coli* strain SE11 isolated from a healthy adult. DNA Res. 2008;15(6):375-86. Epub 2008/10/22. doi: 10.1093/dnares/dsn026. PubMed PMID: 18931093; PubMed Central PMCID: PMC2608844.

59. Iguchi A, Thomson NR, Ogura Y, Saunders D, Ooka T, Henderson IR, et al. Complete genome sequence and comparative genome analysis of enteropathogenic *Escherichia coli* O127:H6 strain E2348/69. J Bacteriol. 2009;191(1):347-54. Epub 2008/10/28. doi: 10.1128/jb.01238-08. PubMed PMID: 18952797; PubMed Central PMCID: PMC2612414.

60. Liu C, Zheng H, Yang M, Xu Z, Wang X, Wei L, et al. Genome analysis and *in vivo* virulence of porcine extraintestinal pathogenic *Escherichia coli* strain PCN033. BMC Genomics. 2015;16:717. Epub 2015/09/24. doi: 10.1186/s12864-015-1890-9. PubMed PMID: 26391348; PubMed Central PMCID: PMC4578781.
